# Elusive ditrysian phylogeny: an account of combining systematized morphology with molecular data (Lepidoptera)

**DOI:** 10.1186/s12862-015-0520-0

**Published:** 2015-11-21

**Authors:** Maria Heikkilä, Marko Mutanen, Niklas Wahlberg, Pasi Sihvonen, Lauri Kaila

**Affiliations:** Finnish Museum of Natural History, Zoology Unit, University of Helsinki, PO Box 17, Helsinki, 00014 Finland; Department of Genetics and Physiology, University of Oulu, PO Box 3000, Oulu, 90014 Finland; Laboratory of Genetics, Department of Biology, University of Turku, Turku, 20014 Finland; Department of Biology, Lund University, 223 62 Lund, Sweden; University of Helsinki, Research Affairs, PO Box 33, Helsinki, 00014 Finland

**Keywords:** Ditrysia, Morphology, Lepidoptera, Phylogeny, Unstable taxa, Total evidence, Deep divergences

## Abstract

**Background:**

Ditrysia comprise close to 99 % of all butterflies and moths. The evolutionary relationships among the ditrysian superfamilies have received considerable attention in phylogenetic studies based on DNA and transcriptomic data, but the deepest divergences remain for large parts unresolved or contradictory. To obtain complementary insight into the evolutionary history of the clade, and to test previous hypotheses on the subdivision of Ditrysia based on morphology, we examine the morphology of larvae, pupae and adult males and females of 318 taxa representing nearly all ditrysian superfamilies and families. We present the most comprehensive morphological dataset on Ditrysia to date, consisting of over 500 morphological characters. The data are analyzed alone and combined with sequence data (one mitochondrial and seven nuclear protein-coding gene regions, sequenced from 422 taxa). The full dataset consists of 473 exemplar species. Analyses are performed using maximum likelihood methods, and parsimony methods for the morphological dataset. We explore whether combining morphological data and DNA-data can stabilize taxa that are unstable in phylogenetic studies based on genetic data only.

**Results:**

Morphological characters are found phylogenetically informative in resolving apical nodes (superfamilies and families), but characters serving as evidence of relatedness of larger assemblages are few. Results include the recovery of a monophyletic Tineoidea, Sesioidea and Cossoidea, and a stable position for some unstable taxa (e.g. Epipyropidae, Cyclotornidae, Urodoidea + Schreckensteinioidea). Several such taxa, however, remain unstable even though morphological characters indicate a position in the tree (e.g. Immidae). Evidence supporting affinities between clades are suggested, e.g. a novel larval synapomorphy for Tineidae. We also propose the synonymy of Tineodidae with Alucitidae, **syn. nov.**

**Conclusions:**

The large morphological dataset provides information on the diversity and distribution of morphological traits in Ditrysia, and can be used in future research on the evolution of these traits, in identification keys and in identification of fossil Lepidoptera. The “backbone” of the phylogeny for Ditrysia remains largely unresolved. As previously proposed as an explanation for the scarcity of molecular signal in resolving the deeper nodes, this may be due to the rapid radiation of Ditrysia in the Cretaceous.

**Electronic supplementary material:**

The online version of this article (doi:10.1186/s12862-015-0520-0) contains supplementary material, which is available to authorized users.

## Background

Five years ago, the backbone branching pattern of the evolutionary tree for one of the largest groups of insects, moths and butterflies (Lepidoptera), was still for the most part unresolved. Our state of knowledge of phylogenetic affinities within this insect order, with over 150 000 described species [1], was well depicted by the comb-like summary tree composed by Kristensen and Skalski [2]. Well-supported hypotheses existed only for the relationships among the most ancient superfamilies. Only very cautious and speculative hypotheses had been proposed for the relationships among the superfamilies of the enormous clade of more advanced lepidopterans, known as the Ditrysia [3, 4]. This ditrysian clade encompasses nearly 99 % of all butterflies and moths [2].

**Fig. 1:**
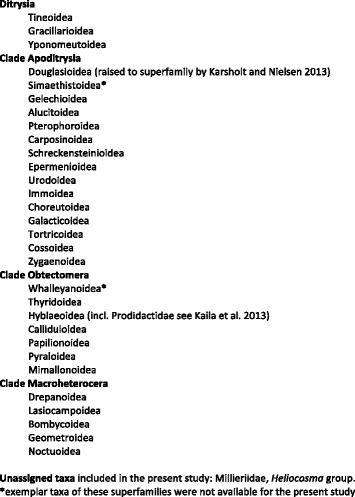
Current classification of Ditrysia, modified from van Nieukerken et al. [1]

Ditrysia are currently divided into 30 superfamilies (classification of van Nieukerken et al. [1], modified by Karsholt and Nielsen [5] who placed Douglasiidae into its own superfamily) (Fig. 1). The size of the superfamilies varies considerably in the number of described species, from several small monotypic superfamilies based on a single genus with only a single or a few species to Noctuoidea with over 40 000 species. Some of these monotypic superfamilies are characterized by features that do not correspond to those diagnosing any of the other superfamilies. The current circumscriptions of the ditrysian superfamilies are based on morphological features and have been generally accepted. Recent studies based on genetic data have corroborated the monophyly of most of the superfamilies [6–12]. However, the interrelationships among most of these superfamilies lack any convincing supporting evidence. This lack of knowledge of evolutionary affinities has hindered further research on this enormous suborder [13].

The initial challenges to inferring evolutionary relationships among the ditrysian superfamilies have been attributed to their morphological homogeneity, and thus to the difficulty of finding distinct, unique morphological characters uniting superfamilies and supporting descent from a common ancestor [2, 13]. Untold numbers of detailed studies on the morphology of Lepidoptera, especially of genital structures and wing venation, have furthered our understanding of the relatedness of species, genera and families, but the relationships among larger assemblages have remained obscure on morphological grounds. The most important studies in comparative morphology aimed at resolving the relationships among ditrysian Lepidoptera were by Brock [14] and Minet [3, 4]. The works by Minet have been influential and the basis for the division of Ditrysia into three nested clades, Apoditrysia, Obtectomera and Macrolepidoptera. These hypotheses are, however, based on a limited number of characters; two for Apoditrysia, two for Obtectomera, and one for Macrolepidoptera. Furthermore, they have not been verified across all ditrysian superfamilies, families or subfamilies.

In recent years, several studies based on molecular methods have brought new information to bear on the evolutionary history among and within the ditrysian superfamilies. These studies range from wide-taxon sampling multigene studies to studies with fewer taxa but with large-scale gene sampling or genomic data [6–12]. The impact of these studies on our knowledge of the relatedness of the lepidopteran superfamilies is beyond question. They have supported some of the previous hypotheses of relationships of ditrysian Lepidoptera, but have challenged or refuted others, e.g. the monophyly of Tineoidea or the position of Papilionoidea. The main division of Ditrysia into the nested clades Apoditrysia, Obtectomera and Macrolepidoptera has been backed to some extent, but with changes. For example, genetic evidence has supported the morphology-based suspicion of Rammert [15] and Kaila [16] that Gelechioidea are a superfamily in Obtectomera to which they were not thought to belong (e.g. [3, 4]). Also, there is mounting evidence that butterflies, skippers and hedylids (together forming Papilionoidea) are more closely related to ‘microlepidopteran’ groups of obtectomeran moths than to the core Macrolepidoptera. The traditional concept of the superfamily Papilionoidea has also changed. The monophyly of Tineoidea has repeatedly been found to be unfounded, and the monophyly of Zygaenoidea has also been challenged as Cyclotornidae and Epipyropidae have not been unambiguously included.

Despite these advances, the evolutionary history of the ditrysian Lepidoptera is still full of questions. The low support values obtained in many studies, especially along the apoditrysian “backbone”, have made the suggested relationships uncertain. A proposed reason for the difficulties in inferring evolutionary relationships among the ditrysian superfamilies is their postulated explosive diversification in the mid- to late Cretaceous into the early Tertiary, contemporaneous with the radiation of flowering plants [17]. When diversification is rapid, little molecular or morphological change has time to accumulate as evidence of common ancestry. This weak signal may also be obscured by subsequent changes in the DNA and morphology. With a limited spectrum of genes it may not be possible to extract this weak signal [18]. To overcome this, phylogeneticists, in lepidopterology as elsewhere, have turned to comparative genomics. By comparing large portions of or entire genomes, clarification of the deeper evolutionary relationships is hoped to be achieved. Recent phylogenomic studies have indeed shown much stronger support values for relationships among the superfamilies than the first studies based on individual gene sequences. However, low taxon sampling still is an issue in these studies. With low taxon sampling, distantly related terminal taxa may group together with strong support values in the absence of more closely related taxa. A wider taxon sampling and more refined models of molecular evolution are needed to overcome this problem [19]. Conflicting hypotheses with strong support values (e.g. the alternative placements of Pterophoridae in Bazinet et al. [6] and in Kawahara and Breinholt [8]) are hard to evaluate if alternative evidence is not available.

Such evidence is traditionally sought in the morphology of the organisms. Synapomorphies, i.e. shared derived characters, are evidence of descent from a most recent common ancestor and inform us of a common evolutionary history. However, as morphology has never been systematically studied across all ditrysian groups, and the data subjected to a rigorous phylogenetic analysis, we lack morphological information of their affinities. The alleged morphological homogeneity of Ditrysia, the laboriousness of comparative morphology, and also the immense number of species of the Order have without doubt held back their classification. Several earlier hypotheses based on morphological features that claim to diagnose certain groups also remain untested across larger groups.

A frequent problem in phylogenetic analyses is taxa that for some reason (rapid rate evolution, lack of data, chimeric terminal taxa - i.e. composed of data from different species) do not find a stable phylogenetic position and may even have a nearly random placement. Such taxa are often called rogue taxa [20]. The presence of such taxa may effectively remove resolution of phylogenetic trees (e.g. [21]). They have also been shown to affect the topology of the entire tree, thus distorting the phylogenetic relationships among other clades [22]. Rogue taxa exert a great influence on the statistical support values of the nodes in phylogenetic trees, obscuring the meaning of these values. Increasing genetic data may not necessarily solve the problem, as fast-evolving taxa may display deviating patterns across large parts of their genomes. Exclusion of rogues is indeed a common practice to try to salvage reliable-looking topologies for the remaining ‘well-behaved’ taxa [23, 24]. Moreover, it is likely that a lot of such pruning goes unreported as it is done during analysis, long before manuscript preparation. Exclusion of rogues, however, means discarding data, and is therefore not an optimal way to treat these taxa, which may be crucial for the study in question. Taxa may behave as rogues in molecular or morphological analyses, or both. However, there is evidence that by combining morphological and molecular data, the negative effect of rogues is reduced and these taxa may find a stable position [25].

Thorough examination of the morphology across all ditrysian groups is also needed to obtain information on character evolution. In a phylogenetic context, morphological structures may show an order of evolution – a polarity. Some characters may also explain the success of certain groups of Lepidoptera. Such characters are also called key innovations as they enable, for example, the exploitation of resources, help avoid predators or survive adverse conditions [26]. The time of emergence of such characters can also be investigated to assess whether certain factors in the geologic history of the Earth have favored Lepidoptera with particular attributes.

Fossil Lepidoptera are extremely hard to identify and assign to particular taxa, because in adults the scale covering hides diagnostic characters, and fossils of soft-bodied larvae are rare [27]. However, a better understanding of the morphological diversity of Lepidoptera and of characteristic features of different groups could prove useful for reliable identification of fossil Lepidoptera. The time of appearance of a certain character is also of interest. Dated and securely identified fossils are used as calibration points in dating phylogenies to obtain estimates of times of divergence [28].

To address these needs, we examine the morphology of ditrysian Lepidoptera across 30 of the 32 currently recognized superfamilies with the aim of providing phylogenetic information on the relationships among them. We examine 530 morphological characters from larvae, pupae and adult males and females of over 300 exemplar taxa. We aimed to obtain samples as comprehensively as possible across lepidopteran subfamilies, and added a number of taxa with unknown or unsupported placement. Special emphasis was placed in the comprehensive inclusion of non-Macrolepidoptera groups and character sampling of all life stages. We have aimed to examine real samples of as many exemplars as possible ourselves. These data have been supplemented and cross-checked using literature sources.

We analyze the morphological data alone and combined with the molecular data set published by Mutanen et al. [9], supplemented with additional taxa and sequences for some taxa. We report the results of the phylogenetic analyses and compare these to the results of other phylogenetic studies on Ditrysia. We re-examine previous hypotheses based on morphology and present new morphological evidence supporting affinities among clades. We also discuss the effects of homoplasy and methodological issues on the results.

## Methods

### Taxa

The exemplar taxa for the study were chosen to represent as many ditrysian subfamilies as possible. We estimate that about 80 % of the 307 subfamilies recognized by Kristensen et al. [13] are included in the present study. An exact number is impossible to give as the concepts of many subfamilies vary or are ambiguously defined. The full dataset consists of 473 exemplar species (Additional file 1). For 52 taxa there are only morphological data, for 153 taxa only DNA data, and for 268 terminal taxa there are both morphological and molecular data. In some cases either the life stages or sequence data were not available from the same species, and so were supplemented by data obtained from a closely related species in the same genus. These cases are indicated in Additional file 1. In addition, 25 non-ditrysian taxa were included in the analyses. All trees were rooted using *Micropterix calthella*. The authors examined the morphology of eight of the 25 non-ditrysian outgroup taxa: *Eriocrania semipurpurella*, *Hepialus humuli*, *Andesiana lamellata*, *Lampronia capitella*, *Incurvaria pectinea*, *Azaleodes micronipha*, *Ptyssoptera sp.* and *Tischeria ekebladella*. The remaining 17 taxa were represented by only molecular data.

The molecular data are largely the same as in Mutanen et al. [9], but excluding *Polypogon strigilatus*, and supplemented by 73 additional taxa and 672 sequences (Additional files 2, 3 and 4). These additional taxa are taxa for which fresh material had become available since the publication by Mutanen et al. or other relevant taxa and data published in previous phylogenetic studies (e.g. [25, 29–31]).

### Examination of morphological characters

Morphological data were collected from 320 species (Additional file 1 lists morphological data for 320 taxa, but in the analyses, data for two species of *Thereutis* were concatenated into one terminal taxon as were those of two species of *Cyclotorna* making the total number of taxa 318). Larval characters were available and coded for 249 of these species, pupae for 245 species, and adult characters for 260 species. When all life stages of same species were not available, character coding was done from a closely related species in the same genus for which material was available. There are seven such cases in our morphological data, indicated in Additional file 1.

Larvae examined were preserved either as dry inflated or in alcohol. Pupae or pupal exuviae were also examined from either dry or alcohol preserved samples, or whenever possible, both. Adult characters were coded from mounted specimens. The wings of the adults were removed and the body treated in 10 % KOH solution to clear it of scales, lipids and muscle tissue. The exoskeleton of the adult was kept in alcohol. The specimens were obtained mostly from the collections of the Finnish Museum of Natural History, but also from several other museums and a large network of international collaborators (see Acknowledgments).

Larvae and pupae were examined for external characters. Due to the paucity of material of the potentially most informative first instar larvae, the larval characters were coded from final instars. Adults were coded mostly for characters of the exoskeleton. Characters known to have much intraspecific variation and/or considerable problems with assessment of homology among more distantly related groups, such as genital structures and wing venation, were not included in the present matrix. Wing venation characters, except for the distinction between homoneurous and heteroneurous venation, were left out from the analyses. Although homologous characters of wing venation are fairly easy to establish for many of the larger taxa, even they posed considerable problems with homologies and continuous nature of variation. These problems were even more pronounced with many groups of smaller moths (over two thirds of taxa are “microlepidoptera”) for which homologies, usually losses of veins and considerable intraspecific variation [32], were impossible to establish. Inclusion of venation characters would have considerably increased the amount of uncertain homologies, thus more likely decreasing rather than increasing the reliability of our morphological data. The head was not opened. The basal wing sclerites were not studied because the observation of the sclerites in the small-sized exemplars would have needed very time-consuming and delicate preparation of special slides. Preliminary exploration of these structures indicated that features of these structures would largely prove continuous, thus a thorough study of them was deemed implausible in the time-frame allocated to the study.

As the focus of the study was to seek morphological evidence to infer phylogenetic relationships among the superfamilies, less attention was paid to intra-family level relationships. Some apparent autapomorphies were coded when they were known to be present in other members of the taxon in question. Literature from which characters for some of the taxa were coded is listed below the character list in Additional file 5. Many of the characters used in this study are not original but were initially taken from literature, and examined across the whole taxon sampling.

Character observations were made solely using light microscopy. A scanning electron microscope would have greatly expanded the number and probably also accuracy of observable characters, but was not available. The observations were made with Leica MZ6, MZ7.5, Leitz Diaplan phase contrast microscope and Wild M10. Character data management and storage was implemented in MorphoBank [33].

The morphological character matrix comprises 530 binary or multistate characters (Additional files 5 and 6). Of these 169 are larval characters, 106 are pupal characters, and 255 are adult characters. Both inapplicable and unclear character states were coded with question marks. Continuous characters were not included in the study. Those typically include characters showing high plasticity, characters based on relative variation in size, shape, or degree of sclerotization, all these being factors that complicate assigning characters into discrete character states. Our focus was to find unambiguous characters suitable as potential diagnostic characters in identification keys.

### Molecular data

Genetic data, totaling 6172 base pairs, were sequenced from 422 taxa. DNA samples were obtained mostly from museum specimens and additional specimens collected during field work in 2009 in New Zealand (Landcare Research global concession permit no. CA5160-OTH) and Tasmania (Department of Primary Industries and Water permit no. FA08264). Genetic data includes cytochrome oxidase subunit I gene (COI) from the mitochondrial genome and seven genes from the nuclear genome; Elongation factor-1a (EF-1a), Ribosomal protein S5 (RpS5), Carbamoyl phosphate synthetase domain protein (CAD), Cytosolic malate dehydrogenase (MDH), Isocitrate dehydrogenase (IDH), wingless, and Glyceraldehyde-3-phosphate dehydrogenase (GAPDH). Sequence lengths, gene summaries and GenBank accession numbers are presented in Additional files 2, 3 and 4, respectively.

DNA was extracted from legs, sometimes from other body parts using Qiagen’s DNeasy extraction kit. DNA amplification and sequencing largely followed the protocol explained in Wahlberg and Wheat [34], but with slight modification and optimization applied to clean-ups of PCR reactions and a different sequencing reaction purification kit (Sephadex G-50, Sigma-Aldrich) applied later. Sequencing was performed mainly with an ABI 3730 capillary sequencer (Oulu), but a smaller part was performed with an ABI PRISMR 3130l capillary sequencer (Turku). Details on DNA extraction are explained in Mutanen et al. [9].

The program Voseq [35] was used to generate sequence data files and gene summaries (Additional file 3).

Matrices were prepared for concatenation in Mesquite [36].

### Analyses

The parsimony analysis of morphological data (318 taxa) was done with TNT Tree search using New Technology Search [37] with the following settings; all four search options (tree fuse, ratchet, random drift & sectorial search) selected; find minimum length 500 times; initial addseq 15; random seed ten. Morphological characters were treated as unordered and equally weighted. Inapplicable, unclear and missing characters were coded with question marks. For examination of trees and character distributions the maximum parsimony trees were exported to Winclada [38], where they were treated as follows: select all trees; keep best trees only. Data sets of pupal, larval characters and adult characters were examined separately and combined. Morphological data were also analyzed with maximum likelihood methods carried out with RAxML [39] in CIPRES [40] using the MK model [41] for morphological data.

DNA data and combined datasets were analyzed using maximum likelihood methods carried out with RAxML [42] in CIPRES [40]. Analyses of combined data based on three different data sets were run:

1. data set of 268 taxa including only taxa for which both DNA and morphological data were available;
2. data set of 422 taxa including all the taxa for which DNA data were available, for 268 of these taxa also morphological data were available and were included, and;
3. data set including all 473 taxa: 268 of these having both molecular and morphological data, 153 taxa with only DNA data and 52 taxa with only morphological data (Additional file 1). Analyses were run with all codon positions retained and with third positions excluded from all genes except EF-1a, which evolves more slowly than any of the other genes [34]. This methodology was also followed in Mutanen et al. [9]. In another set of analyses, both molecular and morphological data were categorized and partitioned according to the rate of evolution of the characters with the programs TIGER [43] and RatePartitions (J. Rota, N. Wahlberg and T. Malm, *in prep*.). Molecular data were divided into seven partitions, morphological data into five. The analyses were run with all partitions present but also with the partitions with the fastest evolving molecular characters removed (partition 7 with 482 characters and partition 6 with 357 characters). Omission of further partitions began to break up well-established clades and produce spurious groupings of taxa. The fastest evolving morphological character partition (partition 5, 42 characters) was also omitted in some trial analyses. However, it became clear that the fastest evolving morphological character partition also contained phylogenetically informative characters and its removal caused false disruptions of even closely related taxa, and was therefore retained in subsequent analyses.

The GTR + G model was used for the DNA data and the MK model [41] for morphological data. The ascertainment bias correction option (Lewis correction) was selected for partitions of morphological data, which include only variable characters. The option to conduct bootstrapping and search for the best-scoring ML-tree in a single program run was selected. Supports for nodes were evaluated with 100–1000 bootstrap replicates. The resulting trees were examined in FigTree (http://tree.bio.ed.ac.uk/software/figtree/).

Mesquite [36] was used to explore character distribution on the trees based on combined data.

We compared our result to other recent studies of ditrysian phylogeny based on molecular data. Comparison of the results with these studies was not straightforward as many of the other studies have much lower taxon sampling and several superfamilies included in the present study are not represented. Comparison of support values obtained for certain clades in different studies is also problematic. We have deliberately retained unstable taxa in our analyses although their presence likely lowers support values, and, especially in consensus trees obtained by parsimony analyses, their presence significantly collapses resolution. To overcome this, and more effectively filter information from the MP trees, we explored them individually.

The character and taxon sampling of the present study were not specifically designed for researching the relationships among subfamilies and families, but we do report on these if they significantly deviate from current concepts. Relationships among non-ditrysian Lepidoptera were not explored.

Many of the morphological characters supporting subfamily, family or superfamily level clades have been found by previous authors and we do not repeat all of them. More detailed information on characters and corresponding references are found in Additional file 5.

## Results

**Fig. 2:**
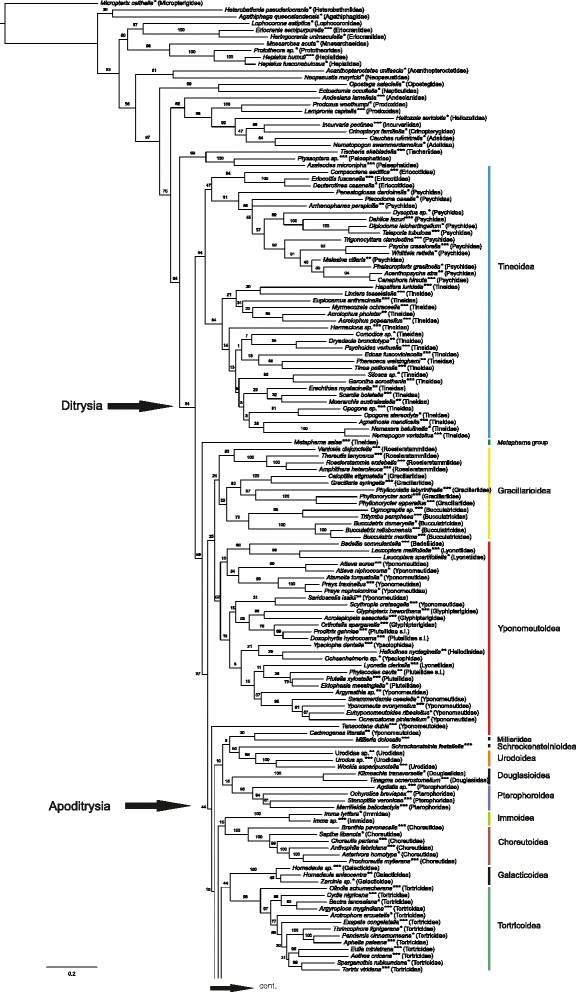
Phylogenetic tree from maximum likelihood analysis of combined morphological and molecular data; 473 taxa; 6702 characters (530 morphological, 6172 bp). ***both DNA and morphological data; **only morphological data; *only DNA data

**Fig. 3:**
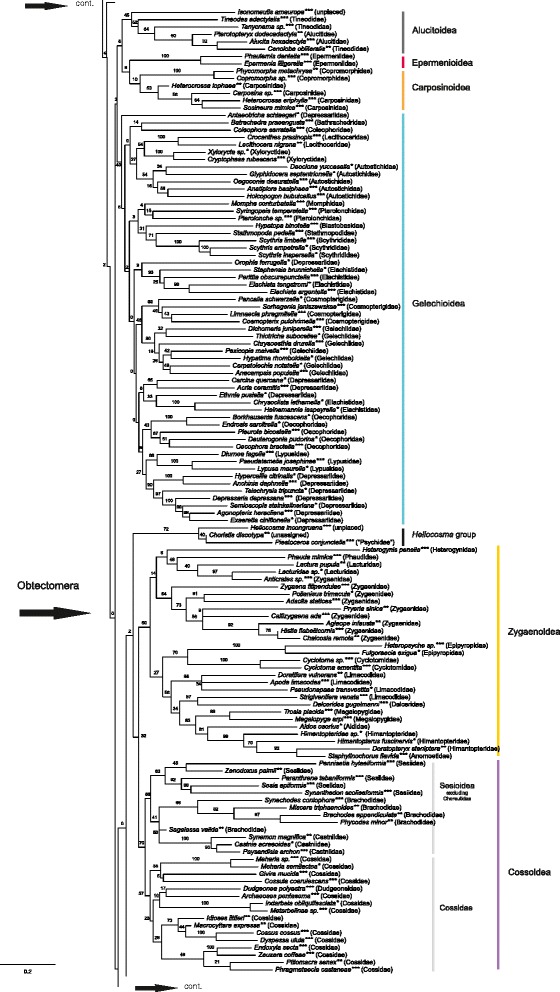
Phylogenetic tree from maximum likelihood analysis of combined morphological and molecular data; 473 taxa; 6702 characters (530 morphological, 6172 bp). ***both DNA and morphological data; **only morphological data; *only DNA data

**Fig. 4:**
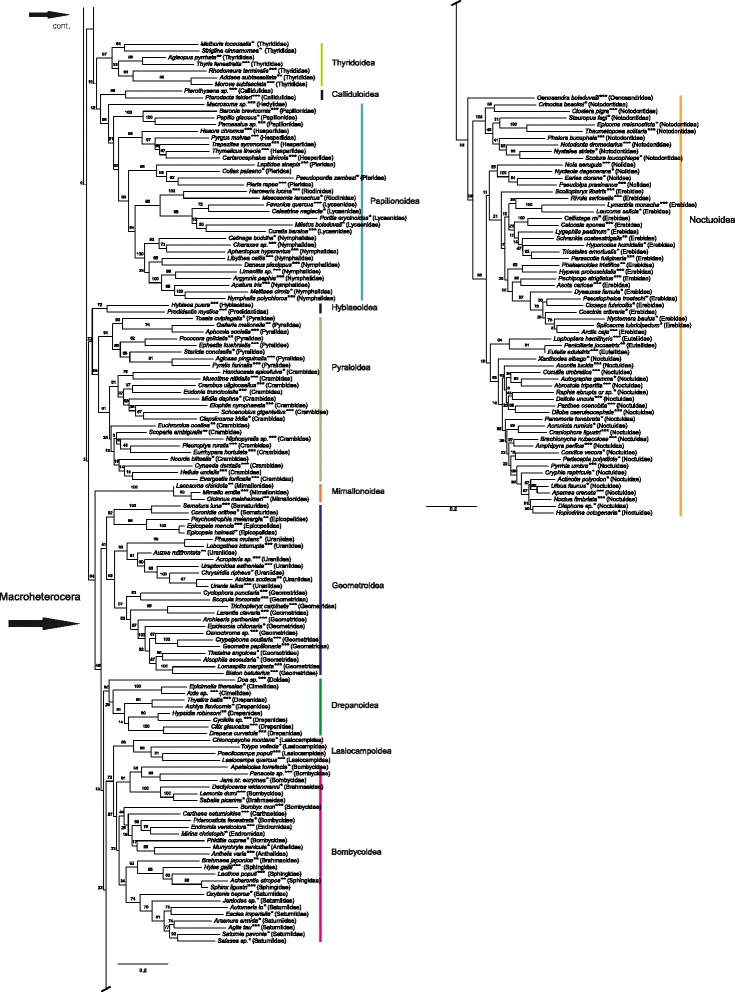
Phylogenetic tree from maximum likelihood analysis of combined morphological and molecular data; 473 taxa; 6702 characters (530 morphological, 6172 bp). ***both DNA and morphological data; **only morphological data; *only DNA data

We report the results of the maximum likelihood (ML) and parsimony (MP) analyses of the morphological dataset alone, and compare these results to those of the ML analysis of combined morphological and molecular data. We consider the total evidence ML analysis (morphology + eight gene regions, with third codon positions retained) (Figs. 2, 3 and 4) our main result. This decision as to the priority of results is based on the principle of inclusion of the maximum amount of data, even though we recognize that this may also bias the results, as may, for example, the inclusion of unstable taxa. Moreover, as Källersjö et al. [44] have shown, third codon positions, even if possibly often saturated, also contain phylogenetic signal that may be informative when combined with other data. We also compare the results with those obtained with third codon positions excluded (Additional file 7) and the “noisiest” molecular character partitions i.e. those with the fastest evolving characters (partitions 7 and 6) removed (Additional file 9). The fastest evolving sites were identified with the programs TIGER, which identifies rapidly-evolving characters (columns in alignment) and RatePartitions, which partitions data according to their rate of evolution (see Analyses). With the fastest evolving molecular character partitions removed, the results were nearly identical to those obtained with all data retained but produced some spurious groupings, which contradict results of other recent studies (see e.g. Additional file 9, Lasiocampoidea nested in Bombycoidea as opposed to them being sister clades, and the presence of the macrolepidopteran clade vs. the macroheteroceran clade as defined in van Nieukerken et al. [1] the latter case of each example obtained in several recent studies based on independent sequence data). In the results of analyses based on data with the noisiest partitions removed, bootstrap support values for the clades were in general lower or more or less equal to those obtained for the same clades in the analyses based on data with noisy characters retained. The results of the analyses based on DNA data alone (Additional file 8) are not discussed separately as they are very similar to those in Mutanen et al. [9], discussed therein.

### Analyses based on morphological data only

The parsimony analysis yielded 19 equally most parsimonious trees (length = 5305 steps, CI = 0.12, RI = 0.62). The resolution of the resulting trees was generally uniform, but interrelationships of certain superfamilies varied among the trees. The strict consensus tree is very poorly resolved due to the adverse effect of some taxa assuming two alternative positions (*Metapherna*, *Ochsenheimeria*, *Euplocamus*, *Heliocosma* group, position of Thyrididae with respect to Mimallonidae and *Hyblaea*, and the alternative positions of Sematuridae, either as sister of Epicopeiidae, or as the sister of Papilionoidea, all these three groups nevertheless in the same monophyletic clade), as well as the paraphyly of Gelechioidea observed in some of the MP trees.

An example of one of the most parsimonious trees is shown in Additional file 10. Most superfamilies are recovered as monophyletic. However, some results appear spurious, such as the consistent grouping of the non-ditrysian *Hepialus* in Tineoidea. Bombycoidea, and in some MP trees also Drepanoidea and Geometroidea, were nested within Noctuoidea. The unstable position of *Ochsenheimeria* is likely due to the internal feeding mode of its larva having caused morphological modifications that remove it from the otherwise constantly recovered association with *Ypsolopha* (Yponomeutoidea), the larvae of which feed externally on leaves of various trees and shrubs. Other differences in the topology were minor rearrangements of apical genera.

The tree resulting from the maximum likelihood analysis of the morphological data set is shown in Additional file 11. There is no support for the deeper nodes, but most superfamilies are recovered as monophyletic except for those in Macroheterocera, which are intermixed.

Separate analyses on data sets of larval, pupal and adult morphology were also run, but are not discussed, because the resulting trees were nearly unresolved due to the small number of characters in comparison to the number of taxa included in these analyses.

### Combined analyses

#### Phylogenetic relationships

The results are in many respects similar to those obtained in the study by Mutanen et al. [9] as the molecular data are largely the same, especially when third codon positions were excluded. Considering that the morphological data in the present study consisted of only 8 % of the total amount of data (10 % when the third codon positions were removed from all genes except EF-1a and 9 % when the fastest evolving molecular character partitions 7 and 6 were removed), the phylogenetic signal from these characters appears disproportionately strong, and their inclusion significantly affects the topology of several clades, while supporting the molecular results elsewhere. As will be discussed in more detail below, morphological attributes stabilized some taxa that had been unstable in DNA-only analyses and thus propose a hypothesis about their phylogenetic affinities. Likewise, molecular data anchored taxa that were unstable in our analyses of morphological data alone. Clades supported by both morphological and DNA-data in general obtained higher support values in the combined analyses than when either type of data was analyzed alone. However, we also obtained some intriguing patterns in the topology that strongly conflicted with the results of several recent studies based solely on genetic data. These patterns show the strength of morphological data over the eight gene regions and the usefulness of different types of data in evaluating different phylogenetic hypotheses. The overall topology of the phylogenetic trees based on the three different data sets (268, 422 and 473 taxa, see Analyses) is very similar and the weakly supported backbone nodes are weak in all trees (Figs. 2, 3, 4 and Additional files 12 and 13). Although the support values are the highest for the smallest data set including 268 taxa (with both DNA and morphological data available for all taxa), and weakest in the trees including all 473 taxa (with either or both types of data), we choose to present the latter (Figs. 2, 3 and 4). The topology of the trees is essentially the same and the tree based on the larger taxon sampling also includes interesting information on the placement and performance of taxa with only either morphological or molecular data. We report support values obtained from all three analyses (268 taxa; 422 taxa; 473 taxa).

## Phylogeny of main ditrysian clades

### Ditrysia

The monophyly of Ditrysia is recovered in the maximum likelihood analyses based on DNA, morphological and combined data sets (Figs. 2, 3 and 4, Additional files 7, 8, 9, 11, 12, 13). The support values for the ditrysian clade in the ML analyses of combined data are as follows: 268 taxa: 99 %; 422 taxa: 85 %; 473 taxa: 84 %. In the parsimony analysis of morphology alone, *Hepialus* is placed among Ditrysia (Additional file 10). Probable reasons for this are that in immature stages there appears to be no morphological characterization that would distinguish Ditrysia from monotrysians, and the methodological decision to exclude morphological characters only applicable for resolving interrelationships within Monotrysia.

The adult characters supporting the ditrysian clade are the name-giving ditrysian female reproductive system i.e. one opening for mating and a separate one for laying eggs [45], and the absence of structures found in some but not all the outgroup taxa such as the division of sternum 2, an elongated metathoracic trochantin, and the tergosternal connection of tergal origin.

### Division of Ditrysia

Our results support previous assertions on the scarcity of clear morphological characters that would define larger assemblages of ditrysian superfamilies [2], and no new such characters were found. Analysis of the morphological data alone or combined with the molecular data set did not improve the resolution of the deeper nodes obtained by Mutanen et al. [9]. Support values for several of the deeper nodes (Figs. 2, 3 and 4), but also of many other nodes, are low. This is in part due to our deliberate decision to not remove unstable taxa from the analyses. The unstable taxa varied depending on the data set and method used. In the maximum likelihood analyses of combined data, the following taxa were unstable: *Imma*, *Millieria*, Douglasiidae, *Tanaoctena*, *Cadmogenes* and several small superfamilies. There were also other taxa with unstable positions, but only within superfamilies, e.g. *Lyonetia* in Yponomeutoidea.

Characters previously proposed by Minet [4] to circumscribe subclades of Ditrysia were tested across the 300+ taxa representing 28 out of 30 superfamilies. The results show that although several obviously contain phylogenetic signal, there is ambiguity in the interpretation and distribution of details of these characters, as already implied by Minet [4].

### Apoditrysia

Characters of the adult sternum 2 have been proposed to define the Apoditrysia, i.e. all Ditrysia except Tineoidea, Gracillarioidea, Yponomeutoidea and Gelechioidea [4, 46]. Recent studies have, however, repeatedly suggested that Gelechioidea belong to Obtectomera, a clade nested in Apoditrysia. Apodemes of sternite 2 of Apoditrysia were defined by Minet [47] to be short, posteriorly enlarged and with anterior corners of sternite 2 distinctly produced laterally. The division between non-apoditrysian and apoditrysian sternum types is also described as the tortricid type versus the tineid type of thoraco-abdominal articulation [46]. The apoditrysian type of sternum 2 is indeed never present in the Tineoidea, Gracillarioidea and Yponomeutoidea, and present in most apoditrysians. However, a condition more similar to the non-apoditrysians is found in some Cossoidea [4], Zygaenoidea [4], Millieriidae [48], Gelechioidea [16, 46], and in the present study, it is observed that clear lateral extensions are occasionally absent even in some Macroheterocera, a clade nested in Obtectomera. In some taxa, sternite 2 is modified due to the presence of hearing organs (e.g. Pyralidae, Uraniidae) making the presence or absence of the lateral extensions difficult to assess. In Mimallonoidea, the presence of the character was also ambiguous.

Sternum 2 has an additional modification that, according to Minet [4], is an autapomorphy for the earlier concept of Drepanoidea (Drepanidae and Epicopeiidae): the lateral extension of sternum 2 is long and reaches in front of the spiracle all the way to the anterior corner of sternite 1. In addition to Drepanidae and Epicopeiidae, this modification is present in Pterophoroidea, *Schreckensteinia, Coleophora* in Gelechioidea, Carposinoidea, Crambidae, Calliduloidea and Papilionoidea (except Pieridae) [14, 47, 49–51].

### Obtectomera

A pupal structure – body appendages (legs and wings) firmly glued to the body (obtect pupa, our character 172) – has been considered a character of considerable phylogenetic significance. A further specialization, i.e. pupa with immobile intersegments between abdominal segments 1 and 4 (our character 231), was used by Minet [3], building upon the work of Chapman [52], to define the clade Obtectomera comprising pyraloids, butterflies and other Macrolepidoptera. In some groups the abdominal segments 1–3 are variably fused, but the mobility of 3–4 (−5) is clear-cut. Therefore the latter was taken into account in character coding. Presence of this pupal type in Yponomeutoidea, Gelechioidea, Epermenioidea and Alucitoidea was interpreted to represent convergence [4]. Now it appears that Carposinoidea (formerly Copromorphoidea), Epermenioidea, Alucitoidea and Gelechioidea belong to Obtectomera sensu Minet [3], so convergence needs only to be assumed for some Yponomeutoidea and possibly for the unstable Immoidea. In the present study, this interpretation is supported when third codon positions or partitions 7 and 6 are removed. With third codon positions included, the Cossoidea + Sesioidea + Zygaenoidea assemblage is nested in Obtectomera, which, on a morphological basis, seems less plausible than the conventional view of them being true non-obtectomerans. The case of Yponomeutoidea is not quite straightforward. The majority of yponomeutoids studied was observed to have the pupa with intersegment 3–4 not entirely either mobile or immobile, but rather having a limited ability for movement. This condition was considered its own character state, supported by experiments where pupal exuviae were softened so that the potential for mobility could be evaluated. Several yponomeutoids with their pupal intersegment 3–4 found entirely immobile are taxa whose thoracic structures largely cover the ventral surface of the abdomen, which alone may effectively prohibit the ability to move abdominal segments and lead to the fusion of the thoracic segments. Immoidea, with an obtect pupa, was associated with Choreutidae, i.e. non-obtectomerans, although with negligible support. The pupal skin in Immoidea is thin, and the level of intersegmental mobility is ambiguous. Minet [4] considered their pupa obtectomeran while Common [53] interpreted it to be of the non-obtectomeran type.

Another character later proposed by Minet [4] to redefine the Obtectomera is the presence of a dorsal lobe or protrusion on the adult pretarsus. As can be noted also from the pictures in Minet [4], the presence of the setose lobe on the pulvillus is not always obvious. The lobe can be small and not easily distinguishable from the non-obtectomeran type of pulvillus, especially if the latter has long setae on the dorsal side. This character was also examined across all taxa in the present study, and the different character states applied here were: pulvillus simple, pulvillus with setose outgrowth, or pulvillus bifid. Setose outgrowths were clearly visible at the base of the dorsal side of the pulvillus in most taxa assigned to Obtectomera. In Gelechioidea, however, the pulvilli were simple. In a few bombycoids and in Pieridae, the pulvillus was also more of the simple type without projecting setae. In other papilionoids, the pulvillus was distinctly bifid and differed from those with a protrusion with setae. *Imma* and *Copromorpha* also had distinct setose lobes on their pulvilli. The resolution limits imposed by the light microscopes used in the present study lowered confidence in coding this character for some of the smaller taxa. In conclusion, our result suggests that even though the Obtectomera is probably largely a valid clade, there are several superfamilies whose assignment to this clade is uncertain, and the degree of homoplasy appears higher than generally presumed. Fänger [54] also examined this character across over 50 ditrysian families with a scanning electron microscope and confirmed its validity with some exceptions.

### Macrolepidoptera and Macroheterocera

The apomorphy proposed to delimit Macrolepidoptera [4], the first axillary sclerite of the forewing with an elongate angle, was not included in the present matrix due to the continuous nature of the character, which compromises its usefulness.

The traditional concept of Macrolepidoptera has recently changed as molecular analyses have repeatedly suggested that Pyraloidea appears to be more close to the core Macrolepidoptera than Papilionoidea and Calliduloidea. The clade including the rest of the traditional Macrolepidoptera (Lasiocampoidea, Bombycoidea, Drepanoidea, Geometroidea and Noctuoidea) is referred to as Macroheterocera [1]. In several recent molecular analyses, Mimallonoidea has been placed as the sister-group of Macroheterocera. This is also the result obtained in our analysis with the three codon positions included (Figs. 2, 3 and 4). In analyses without the third codon positions or fast evolving partitions (Additional files 7 and 9), the pattern changes and the arrangement of clades varies, with all alternatives having low support.

Although the macroheteroceran clade is recovered in the combined analysis, no unique morphological character was found to unite them.

### Interrelationships and composition of ditrysian superfamilies

The most basal clade of Ditrysia recovered in the combined analysis (Figs. 2, 3, 4 and Additional files 12 and 13) is a monophyletic Tineoidea with the following support values: 268: 88; 422: 65 and 473: 54 %. They are also monophyletic in the result based on DNA with all three codon positions retained and those of combined data with the fastest evolving partitions removed (Additional files 8 and 9). Tineoidea are divided into two clades. One clade includes the monophyletic families Eriocottidae and Psychidae (support 268: 87; 422: 40; 473: 47 %), the other Tineidae (support 268: 100; 422: 100; 473: 64 %). In recent phylogenetic studies based on DNA data alone, a monophyletic Tineoidea has only been obtained in the studies by Regier et al. [10] and [55] in analyses of unaltered nucleotides from all three nucleotide positions (nt123). In the present study, when the third codon positions are removed (Additional file 7), Tineoidea are paraphyletic, a result similar to that obtained in other recent studies based on sequence data [6, 9, 10, 55]. Tineoidea are then paraphyletic, with Eriocottidae, Psychidae, and Tineidae as separate clades. The present study did not include exemplar species of Meessiidae (in its revised concept), included in the phylogenetic study on Tineoidea by Regier et al. [55]. In the results of their study, representatives of Meessiidae were excluded from all previously recognized tineoid families and placed as sister to all other Tineoidea + Ditrysia, thus also contributing to the paraphyly of the superfamily. Had Meessiidae been included in the present study, the monophyly of the superfamily could have been challenged.

**Fig. 5:**
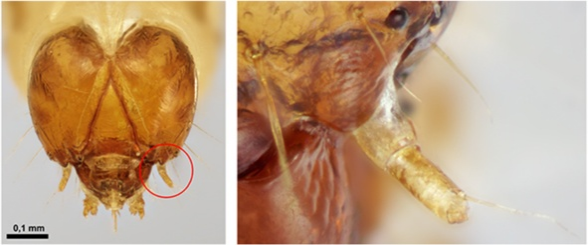
Unique tineid apomorphy: more or less triangular cap dorsally covering base of larval antenna. *Morophaga choragella*

Tineoidea were also recovered as monophyletic in the maximum likelihood analysis of morphological data (Additional file 11), but not in the parsimony analysis (Additional file 10). Adults in all three tineoid families included in the present taxon sampling are characterized by the presence of a distinctly shortened proboscis with loose galeae (or complete absence of the proboscis) and pseudapophyses found in most females [56, 57]. Shortening or absence of galeae is, however, found throughout the ditrysian superfamilies. The looseness of galeae could not be coded because most of the short galeae in other groups also loosened when treated in KOH solution, and on non-denuded specimens the scale coverage effectively prohibited the reliable observation of this character. Light microscopes were insufficient for comparison of differences in the microstructures holding galeae together. No support for the monophyly of Tineoidea was found from larval or pupal characters. Within Tineoidea, monophyly of Tineidae is supported by a unique synapomorphy, a nearly triangular cap dorsally covering the base of the larval antenna (Fig. 5). *Dryadaula*, recently proposed to be a new family, Dryadaulidae Regier et al. [55], is nested in Tineidae, possibly because only morphological data from the adult male and female were available and the larval synapomorphy was not studied. Psychidae larvae share the median fusion in all thoracic legs; the members of the subfamily Oiketicinae are characterized by the horizontal position of the larval spiracles of T1.

*Metapherna* Robinson & Nielsen [58] is an Australian genus that has been placed in Myrmecozelinae in Tineidae, although Robinson & Nielsen [58] noted that it has, e.g., an unusually long proboscis for a tineoid. In the analyses of DNA data-only and of combined DNA and morphological data (268: 89; 422: 64; 473: 59 %), *Metapherna salsa* (Meyrick, 1920) is placed between Tineoidea and the rest of Ditrysia (Figs. 2, 3, 4, Additional files 7, 8, 9, 12 and 13). In the morphology-only ML analysis *Metapherna* has an unresolved position within non-apoditrysians (Additional file 11). In the MP analysis it is placed either as a basal ditrysian or in Yponomeutoidea, but never in the Tineoidea (Additional file 10). Based on genetic evidence and their divergent morphology, species of the *Metapherna* group [listed in Robinson & Nielsen [58]] obviously may eventually require their own family.

In the analyses of combined data (Figs. 2, 3, 4, Additional files 12 and 13), *Metapherna* is basal to a large clade (268: 85; 422: 57; 473: 37 %) divided into a rather weakly supported clade including taxa assigned to Yponomeutoidea and Gracillarioidea (268: 46; 422: 50; 468: 35 %), and the apoditrysian clade (268: 43; 422: 49; 473: 44 %). Yponomeutoidea plus Gracillarioidea, with Douglasiidae excluded, have also been found to be monophyletic in several other recent phylogenetic studies on the phylogeny of Ditrysia [6–12], and also in two studies focusing on the phylogenetic affinities within these superfamilies [59, 60]. In analyses based on morphology only, Douglasiidae was included in Gracillarioidea, as has also been formerly suggested [61] (Additional files 10 and 11). However, in our preferred result (ML of combined data, Figs. 2, 3 and 4) it is associated, with uncertain position, among Apoditrysia, as in those recent molecular studies in which it has been included.

**Fig. 6:**
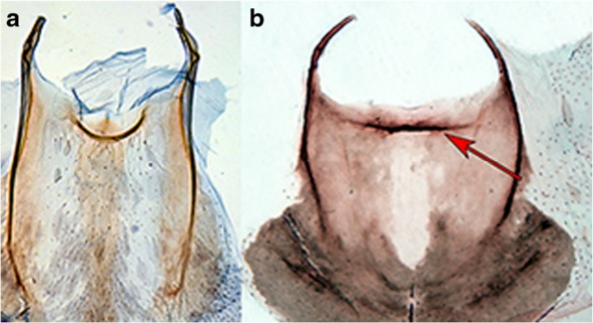
Transverse costa behind anterior margin of sternum II. **a** *Yponomeuta evonymellus* (Yponomeutoidea); **b** *Roeslerstammia erxlebella* (Gracillarioidea)

In the present study, we did not find any unique characters common to all members of Gracillarioidea and Yponomeutoidea. This grouping was supported by a combination of homoplasious characters such as characters of antennal scaling and the tendency to have the proximal pair of tibial spurs of the hindleg on the proximal half of the leg. However, the transverse costa at the base of sternum A2 found in most Yponomeutoidea and proposed to be a possible groundplan autapomorphy for the superfamily [62] was also found to be present in Roeslerstammiidae (Gracillarioidea): *Roeslerstammia* (Fig. 6), *Vanicela*, *Amphithera* and the female of *Thereutis*. A similar costa, although longer and often reaching the sternal apodemes, was present on sternum 2 of Choreutoidea (see Fig. 1 in Rota and Kristensen [48]). Some tortricids also exhibited a sclerotized bar or patch in a similar position.

Yponomeutoidea were found to be monophyletic (268: 31; 422: 34; 473: 52 %) and the presence of the pleural lobes on sternum 8 of the males was verified. Pleural lobes were found to be present in nearly all yponomeutoids. In some cases, the expansion of the lobe was not strong and it was difficult to determine if lobes were present. They were absent in *Cadmogenes literata* (Meyrick 1923), which is currently placed in Plutellidae, but may eventually need to be placed in its own family [63]. In our study, only morphological data were available for *Cadmogenes.* Its position was unstable, and it grouped most often with some other unstable taxa, such as *Millieria* or *Tinagma*. As in previous studies, the position of *Lyonetia* was unstable within Yponomeutoidea. Intriguingly, it was rarely associated with *Leucoptera* (Cemiostominae), which has been considered a subfamily of Lyonetiidae. This result is in line with that obtained by Sohn et al. [60]. In our result Heliodinidae (only morphological data available) was grouped together with *Ypsolopha* and *Ochsenheimeria*, which deviates from all former suggestions, where it most often is considered close to Bedelliidae [60]*.*

Gracillarioidea (268: 10; 422: 18; 473: 24 %) had no distinct immature or adult characters common to all taxa in the character sampling of the present study.

Several small superfamilies, families and taxa, the position of which has greatly varied in analyses based on genetic data — Millieriidae, *Tinagma*, *Tanaoctena*, Epermenioidea, Immoidea, Carposinoidea, Pterophoroidea, and Choreutoidea — do not find a stable position in the combined analysis either. They tend to form one or two agglomerations, or join other groups with extremely low support values. A repeatedly recovered pattern is that Alucitoidea, Epermenioidea and Carposinoidea form a monophylum. However, interesting observations were made regarding these taxa. Characters listed below could prove useful in diagnosing families even though they may not have phylogenetic signal at higher levels. For example, the sculpture of the larval mentum is dentate in two groups, viz. Choreutidae and Alucitoidea s. l., respectively. The larvae of Carposinidae have a characteristic bilobed extension between the pre- and submentum.

In the analyses based on molecular data (Additional file 8) only, *Schreckensteinia* is an unstable taxon, taking multiple alternative positions in the resulting phylograms. In the combined analysis, *Schreckensteinia* consistently groups with the Urodidae (Figs. 2, 3, 4, Additional files 12 and 13; 268: 56; 422: 60; 473: 50 %). They share several, yet to some extent homoplastic, synapomorphies in their larvae, notably the closely set stemmata, granulose sculpture of the mentum, medially narrowed prolegs with mesoseries on A3-6, and the closely set prolegs on A10. The adult *Urodus* (Urodidae) has pigmented patches on the cuticle of its mesothorax, which are also found in Roeslerstammiidae (Gracillarioidea).

Immidae have some obtectomeran features such as the setose outgrowth on the dorsal side of the pulvilli [54], and a possibly obtectomeran pupa (see above). Immidae have previously been placed in Sesioidea [64] and as an unplaced obtectomeran superfamily of their own [65].

Alucitoidea are monophyletic and are joined by *Isonomeutis amauropa* Meyrick, 1888 in the combined analysis (Figs. 2, 3, 4, Additional files 12 and 13; 268: 57; 422: 36; 473: 45 %)*. Isonomeutis* has previously been placed in Copromorphidae. In our analysis, both with and without the third codon positions or fast evolving partitions removed, Alucitoidea, Carposinoidea and Epermenioidea are grouped together (Additional files 7 and 9). The family Alucitidae is consistently nested in Tineodidae. A tongue-shaped lobe laterad of the larval submentum is an autapomorphy for *Isonomeutis* + Alucitidae + Tineodidae. On the basis of this evidence (268: 85; 422: 77; 473: 52 %), we here synonymize Tineodidae with Alucitidae, **syn. nov.**

The adult character common to Pterophoroidea, here only including Pterophoridae, is the acute angle of the metepimeron [4]. However, the metepimeron of *Tanycnema anomala* (Tineodidae) is also rather acute. Pterophoroidea also have the prespiracular extension of sternum 2 discussed above in the section on Apoditrysia.

Galacticoidea are consistently recovered as the sister group to Tortricoidea (268: 81; 422: 55; 473: 44 %), which is also found in the results of the ML analyses of morphology (Figs. 2, 3, 4, Additional files 11, 12 and 13). Galacticidae also group with Tortricidae in Regier et al. [10]. Tortricoidea are monophyletic in all analyses. Tortricoidea have been characterized by the flat papillae anales of the females [66]. However, similarly flat papillae were occasionally also present in several other groups, e.g. Zygaenoidea, Pyraloidea, *Prodidactis*, Calliduloidea and Noctuoidea. These taxa, however, have attributes that suggest the character has evolved repeatedly.

The *Heliocosma* group was removed from Tortricidae by Horak and Common [67], and left without family assignment. In the study by Mutanen et al. [9] it was, together with another unassigned taxon, *Piestoceros*, grouped with Brachodidae. Regier et al. [10] recovered *Heliocosma* together with Tortricoidea. In our result of the analysis with three codon positions included, *Heliocosma, Piestoceros* and *Choristis* form a monophylum that, with very weak support, forms the sister-group of the Cossoidea + Sesioidea + Zygaenoidea assemblage (Figs. 2, 3 and 4). With the third codon positions removed, they are associated with Immoidea (Additional file 7). When the fast evolving partitions are removed, they group with *Millieria* (Additional file 9). This group is thus one of the most unstable ones in Lepidoptera. No hypothesis of its position can be preferred over others at present and morphology does not offer clear clues about its affinities either.

Zygaenoidea (268: 79; 422: 76; 473: 60 %), Sesioidea (Choreutidae excluded) (268: 57; 422: 19; 473: 56 %) and Cossoidea (268: 43; 422: 25; 473: 57 %) are all, respectively, found to be monophyletic in our results and are grouped together in the same clade (268: 55; 422: 36; 473: 32 %) (Figs. 2, 3, 4, Additional files 12 and 13). In the smallest data set (268 taxa) *Synechodes coniophora* (Brachodidae), however, is placed within Cossidae. No other brachodids were included in the analyses of the 268 and 422 taxa data sets due to the unavailability of DNA data. In the results based on DNA data only Zygaenoidea are monophyletic (except for Epipyropidae and Cyclotornidae), but Sesiidae and Cossidae are intermixed (Additional file 8). In other recent studies based on genetic data, Sesiidae and Cossidae have not been found to be monophyletic either [7, 9–11]. Sesiidae and Cossidae are also considered to belong to the same superfamily according to the most recent classification of Lepidoptera [1].

**Fig. 7:**
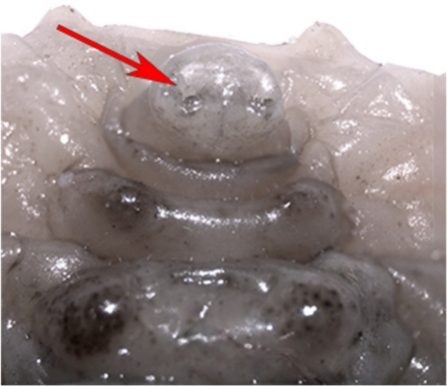
*Cyclotorna* larval segment A10, ventral view, showing swelling on which both prolegs are positioned

Epipyropidae and/or Cyclotornidae were not included in Zygaenoidea in Mutanen et al. [9], Cho et al. [7], Regier et al. [10] or Bazinet et al. [6], but in the present study belong there with moderate support (268: 79; 422: 76; 473: 60 %). The support seems largely due to synapomorphies of immatures shared with at least some other Zygaenoidea: prolegs of larval A10 are approximate and arise on a common protuberance (Fig. 7); the spiracle of abdominal segment 1 of pupae is exposed (not always in Epipyropidae, for example in our exemplar taxon *Heteropsyche*), and there is a sculpted, laterally extended flange on the pupal eyepiece (see Epstein [68] for explanation). Species in both families are fully or partially parasitic at the larval stage. There are indications that molecular evolution of parasitic species may often be accelerated [69], possibly explaining the long branches and unstable phylogenetic affinities of Epipyropidae and Cyclotornidae in molecular data sets. This is also the first evidence that epipyropids and cyclotornids might be sister groups (268: 69; 422: 74, 473: 70 %).

Minet [4] proposed the presence of two asymmetrical pits on the lower frontoclypeus of adults as a possible autapomorphy for Cossidae. Such pits were present in several, but not all cossids. At times, it was difficult to tell the difference between such pits and large scars of scales i.e. the round sockets in which scales were attached, on the frontoclypeus. Better magnification would be necessary to observe the details.

In the analyses based on molecular data only, Sesioidea are not monophyletic, a result also found in Mutanen et al. [9]; Regier et al. [10], Cho et al. [7] and Bazinet et al. [6]. In the combined analysis, however, they are (except for the sole representative of Brachodidae in the 268 data set, see above) though with low bootstrap support (268: 57; 422: 19; 473: 56 %; Figs. 2, 3, 4, Additional files 12 and 13). *Pennisetia* (Tinthiinae)*,* which in our DNA-only analyses was not with Sesiidae, joins other sesiids because it shares several adult characteristics with them, such as the sesiid wing-locking mechanism and a Y-shaped suture/depression on the mesepimeron [70]. Adult characters common to Brachodidae, Sesiidae and Castniidae are the elongated posterior tendons of the metafurca (mentioned as a possible synapomorphy for the three families by Minet [3]) and the extension on the metafurca [14]. Several females in these families also have a telescopic ovipositor. The immature stages of sesioids and cossoids are generally uniform, suggesting no obvious support for the monophyly of Sesioidea.

The anteriorly more heavily pigmented ocular diaphragm was suggested as an autapomorphy of Sesioidea [4]. We were not able to examine the pigmentation of the ocular diaphragm, because of the varying success in treating the samples in KOH solution so that the diaphragm would be sufficiently clearly visible. The size of the patagia, which has been mentioned as a synapomorphy of Sesiidae, Castniidae and Brachodidae, is a continuous character with much variation among Lepidoptera and therefore was not included in the present study.

In addition, *Paysandisia* (Castniidae) shares the character considered autapomorphic for Sesiinae, the bag-like protuberance concealing the pleural suture [70].

In our result, Cossoidea + Zygaenoidea + Sesioidea are in Obtectomera. The support for this finding is, however, negligible and disappears when third codon positions are removed (Additional file 7). Several recent studies present evidence for the exclusion of these from Obtectomera. Our result is nevertheless intriguing as a similar pattern with the Cossoidea + Zygaenoidea + Sesioidea assemblage nested within Obtectomera has also been recovered by Regier et al. [11] in some of the analyses based on five protein-coding genes. If this pattern indeed proves the most likely, it emphasizes the level of convergence in morphology (see also Carter and Kristensen [71]). In the morphology-only analyses, the superfamilies are not in Obtectomera.

**Fig. 8:**
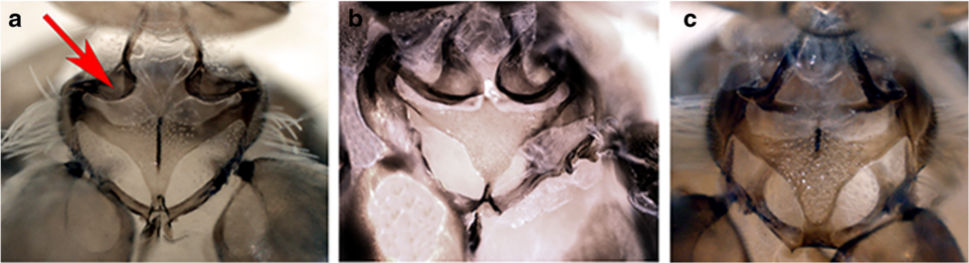
Cervical sclerites. **a** *Anthophila fabriciana* (Choreutidae); (**b**) *Brachodes appendiculata* (Brachodidae); (**c**) *Cydia nigricana* (Tortricidae)

Choreutidae is a family without known phylogenetic position. Choreutidae had previously been placed in a number of different families until Minet [4] moved them to a superfamily of their own. In our results, Choreutoidea are monophyletic, and are placed in a very weakly supported clade together with Immoidea. In the result obtained without third codon positions (Additional file 7), the placement of Choreutoidea shifts to a monophylum together with Galacticidae and Tortricoidea, a finding also recovered by the MP and ML of morphological data (Additional files 10 and 11). The morphological data collected from the choreutids in the present study contain, however, intriguing features. The cervical sclerites of the adult prothorax are very similar to those of Brachodidae as being strongly turned inwards in both groups (Fig. 8). In brachodids, the lower arm of the sclerites is also shortened. Similar, but not as strongly turned cervical sclerites were present in Castniidae and some Cossidae. Other Lepidoptera do not have such wide and inwardly turned cervical sclerites (e.g. Tortricidae in Fig. 8). Brock [14] and Heppner [64] have proposed a close affiliation of these families but based on characters not included in our study (wing venation and genitalia). The dentate sculpture of the larval mentum characterizes Choreutidae, but also the *Isonomeutis-*Tineodidae-Alucitidae clade, which does not seem closely related to Choreutidae.

Millieriidae are a small microlepidopteran family, which was formerly considered a subfamily in Choreutidae [72]. Heppner considered Millieriidae to be “somewhat of a missing link between other Choreutidae and the remainder of the Sesioidea”. Based on evidence from morphological and molecular studies [9, 73–75] Millieriinae were excluded from Choreutidae and elevated to family level by Rota [75]. Rota and Kristensen [48] compared the thoraco-abdominal articulation of Millieriidae to those of Choreutidae and found that unlike choreutids, Millieriidae do not have lateral extensions on the apodemes on sternum 2. In the study by Mutanen et al. [9] *Millieria* was placed together with the unstable Douglasiidae. In the present study, *Millieria* is grouped, with weak support, in a clade that contains *Cadmogenes*, Schreckensteinioidea and Urodoidea (Figs. 2, 3 and 4). In morphology-only analyses, *Millieria* is associated either with Choreutidae or Roeslerstammiidae (Additional files 10 and 11). The former position may be explained by the external resemblance of the adult of *Millieria* to Choreutidae in having a scaled base of the proboscis and scales between the eye and antenna. However, both features are also found in other groups. The immature stages of *Millieria* give little information. The larva is an internal feeder, and devoid of obvious traits that could help group it with other taxa. Its non-obtectomeran pupa is also generalized in appearance, thus not providing data for phylogenetic insights, either. The position of Millieriidae with Roeslerstammiidae is based on very homoplasious characters, such as the external invisibility of sutures between pupal abdominal segments 8–10, thus without convincing morphological support.

The affinities of Papilionoidea have varied among recent molecule-based studies. Common to all of them is that the superfamily appears to be closer to the ‘microlepidopteran’ grade instead of being among the core Macrolepidoptera as has been the conventional view. Another pattern repeatedly recovered in studies based on sequence data [8–10, 12] and a study combining molecular and morphological data [29], has been that Papilionidae is the sister of the other papilionoid families, including Hedylidae and Hesperiidae. In the present study, a surprising result is the emergence of a topology with the conventional concept of Papilionoidea, i.e. Hedylidae as the sister group of the rest of Papilionoidea (Figs. 2, 3, 4, Additional files 12 and 13; 268: 84; 422: 61; 473: 51 %). The position of Hesperiidae depends on the data set analyzed and on the inclusion or exclusion of third codon positions. If these are excluded, Hedylidae are still the most basally arising group of papilionoids, but Papilionidae and Hesperiidae shift places (Additional file 7). This result is in conflict with the recent studies based on genetic data in which both Hedylidae and Hesperiidae are nested within Papilionoidea.

The closest relatives of Papilionoidea are not recovered with unequivocal support in recent molecular studies. The present result suggests that Callidulidae is a sister-group to Papilionoidea (268: 52; 422: 48; 473: 42 %), and if third codon positions are removed, Callidulidae are sister to Papilionoidea + Macroheterocera.

A monophyletic Gelechioidea is recovered in the combined (268: 90; 422: 34; 473: 40 %) and morphology-only ML analyses. The adult character supporting the grouping is the scaled haustellum. The character is not unique to Gelechioidea, being also found in Tischerioidea, Pyraloidea, Choreutoidea and Millieriidae. A monophyletic Gelechioidea has been found in all recent molecular studies on ditrysian phylogeny and targeted studies on Gelechioidea [6, 7, 10–12, 16, 25, 30]. Although Gelechioidea are found to be monophyletic, support values for the clade are never very high when more than just a few exemplar species are included in the analyses. The large phylogenetic studies based on genetic data have also shown that Gelechioidea belong to the obtectomeran clade, contradicting the earlier assumptions of their affinities with the non-apoditrysian Yponomeutoidea [4].

The closest relatives to this massive radiation of small moths are not known. The results of the present study do not give unambiguous clues to resolve this. Several recent studies have recovered a sister relationship between Gelechioidea and Thyridoidea [6, 12] and in Kawahara and Breinholt [8] they are sister to the clade Callidulidae + Thyrididae. Our study suggests, though with negligible support, a sister group relationship between Gelechioidea and a clade comprising Alucitoidea, Epermenioidea and Carposinoidea (Figs. 2, 3, 4 and Additional file 9), or if third codon positions are excluded, a sister group position to all other Obtectomera (Additional file 7).

In our results, Hyblaeoidea (including Prodidactidae, see Kaila et al. [76]) are the sister-group of Pyraloidea (268: 68; 422: 63; 473: 72 %) (Figs. 2, 3, 4 and Additional files 9, 12 and 13). In the results based on the 268- and 422- taxon data sets, *Copromorpha lichenitis* joins Hyblaeoidea (268: 28; 422: 29 %) whereas in the 478-taxon set it is associated with 100 % support with *Phycomorpha metachrysa*, another copromorphoid, and together they weakly form a clade with Carposinidae, currently also placed in Copromorphoidea. The grouping of *Hyblaea* and *Prodidactis* is supported by a membranous projection present on the coxae of the male and a modification of the apex of the larval spinneret, as has been explained in Kaila et al. [76]. Such a modified larval spinneret is also present in Thyridoidea and several papilionoids, including all Hesperiidae examined. Coxal processes were also found in several species in Geometridae, in Cyclidiinae (Drepanoidea), and in *Anchinia daphnella* (Gelechioidea). The affinity of *Prodidactis* to several other families has previously been investigated by Epstein and Brown [77] with inconclusive results, and the genus was assigned a family of its own. Based on the two above-mentioned characters in common with *Hyblaea* and congruent evidence from five genes, Kaila et al. [76] placed Prodidactidae in Hyblaeoidea. In the present study, the results of the analysis based on combined data and those based on DNA data only, the clade formed by *Hyblaea* and *Prodidactis* tended to group basal to Pyraloidea (268: 18; 422: 8; 473: 22 %; Figs. 2, 3, 4 and Additional files 7, 9, 12 and 13). Morphological characters supporting this position are not clear. The papillae anales of the female of *Prodidactis* are flat, as they are in several pyraloid species. In the maximum likelihood analysis of morphological data, *Hyblaea* and *Prodidactis* also group together, their position being close to Mimallonoidea and Thyridoidea. Apart from the present study and Mutanen et al. [9], Hyblaeidae and Prodidactidae have not been present in phylogenetic studies based on genetic data.

Thyridoidea are recovered as monophyletic with strong support (268: 93; 422: 84; 473: 87 %). Thyrididae are a somewhat mobile group in our analyses, but often group as sister to Callidulidae and Papilionoidea. We found the adult character proposed by Minet [4] to support the monophyly of the superfamily, the lateral lobe on A1, very difficult to observe. Out of our five exemplars of Thyridoidea, the character was more or less clearly present only in *Thyris fenestrella*. Due to the difficulties in identifying this character, it was not included in our matrix. However, a number of thyridoid species showed on abdominal segment A1 a structure similar to the postspiracular bar found in Papilionoidea. The presence of this bar could associate thyridoids in the same clade with Papilionoidea, a result also observed in the molecular analysis by Mutanen et al. [9]. In most samples of Papilionoidea, the postspiracular bars are strongly sclerotized and extend diagonally from the posterior edges of tergum 1 towards the anterior edge of sternum 2. The bar may be long and reach behind the spiracle, but sometimes short and harder to define. In addition, the bar is not always easy to distinguish from other sclerotized, but more perpendicularly directed, extensions of the posterior edge of tergum 1 or the anterior edge of tergum 2 reaching toward the sternum. Several of the female Thyridoidea have fine hair-like bristles by the hindwing acanthi in addition to the usual stouter bristles. In other recent studies the closest relatives of Thyridoidea have varied, but they seem most often to group either close to Papilionoidea, Gelechioidea, Calliduloidea, Pterophoroidea or other small apoditrysian families (Alucitidae, Hyblaeidae, Carposinoidea) [6–12].

Pyraloidea are monophyletic with strong support (268: 88; 422: 98; 473: 95 %). The most obvious adult characters supporting the clade are the scaled haustellum and the characteristically-shaped abdominal hearing organs. Differences in the hearing organs separate Pyralidae and Crambidae [47]. The sclerotized structures of the tympanal organs have been regarded as homologous in Pyralidae, Crambidae, Dudgeonidae, female Uraniidae and Geometridae because they are modified abdominal apodemes [78–80]. A monophyletic Pyraloidea, with monophyletic Pyralidae and Crambidae, have also been found in all studies based on genetic data in which representatives of both families have been present [6, 7, 9–12].

Mimallonoidea (3 exemplar species) are placed as the sister-group of the Macroheterocera (Drepanoidea, Lasiocampoidea, Bombycoidea, Geometroidea and Noctuoidea) (473: 54 %). This position has also been obtained in several other studies based on genetic data (although not in the Bayesian analysis in Mutanen et al. [9] and in the ML analysis of Cho et al. [7]). In Regier et al. [10], Mimallonidae are in a clade with *Axia* (Cimeliidae) and *Doa* (Doidae) and together form the sister-group of Drepanidae. No particular character in the adult morphology explains this position as the sister-group of Macroheterocera. In the morphology-only analyses Mimallonoidea are monophyletic but are associated with Thyridoidea and Hyblaeoidea (Additional files 10 and 11). In the results based on the 268- and 422-taxon sets, *Mimallo amilia*, as sole representative of the superfamily in these analyses, is placed as sister to Bombycoidea or Lasiocampoidea (268: 49; 422: 58 %).

The Macroheteroceran clade (268: 96 % (but including Mimallonoidea); 422: 99 % (including Mimallonoidea); 473: 48 % (excluding Mimallonoidea)) is one of the most strongly supported clades, but the support values for the relationships among the superfamilies are not strong. The obtained topology (Figs. 2, 3 and 4) is as follows: (Geometroidea s.l.) + (Drepanoidea + ((Lasiocampoidea + Bombycoidea) + Noctuoidea)). The macroheteroceran superfamilies are recovered monophyletic in analyses based on or including genetic data (Figs. 2, 3, 4, Additional files 7, 8, 12 and 13; when fast evolving partitions are removed (Additional file 9) Lasiocampoidea are nested in Bombycoidea), and the monophyly of most is supported by morphology (see e.g. [81–83]). However, in morphology-only analyses the phylogenetic signal of these morphological traits does not suffice to recover the clades and they are intermixed (Additional files 10 and 11). The proposed relationships among the superfamilies cannot either be unequivocally explained on morphological grounds. In recent molecular studies, the proposed relationships among the macroheteroceran superfamilies vary [6–12].

In the result of the analysis of combined data Geometroidea s. l. is obtained (473: 41 %). Sematuridae and Epicopeiidae are sister groups and together form the sister group of Uraniidae + Geometridae (Figs. 2, 3 and 4). The obtained monophyly and within superfamily relationships are identical to those recovered in a very recent study based on molecular data on the phylogeny of Geometroidea [84], which, however, are based on subsample of the present molecular dataset. Rajaei et al. [84] also characterized a new geometroid family, Pseudobistonidae, based on *Pseudobiston pinratanai* Inoue, 1994, not included in the present taxon sampling. In their study, Pseudobistonidae is placed as sister-group of Epicopeiidae.

Rajaei et al. [84] proposed five apomorphies supporting the monophyly of Geometroidea. Two of these are wing characters not included in the present study. The authors acknowledge that the other three characters are not clear-cut features. Only one of these characters, the reduction of the fenestrae laterales, was included in our study. Although the character is not always easy to define and may also be found in other Ditrysia, our observations are generally in agreement with those by Rajaei et al. A list of characters regarded as synapomorphies supporting the relationships among families within Geometroidea is provided by Rajaei et al. [84]. Several of these characters are continuous in nature and are not included in the present study.

In the present study, the monophyly of Geometroidea is, however, occasionally broken in the analyses of the 268- and 422-taxon sets and when the third codon positions or the two fastest evolving partitions are removed as Sematuridae + Epicopeiidae are placed in a position distant from the rest of Geometroidea (Additional files 7, 9, 12 and 13). Epicopeiidae, traditionally placed in Drepanoidea [83, 85], also group with Sematuridae in Regier et al. [10, 11], and are sister to the Uraniidae + Geometridae clade. In Cho et al. [7] Epicopeiidae and Sematuridae are also sister-groups in the nt123 partitioned analyses, but not in the “all non-synonymous” analyses. Bazinet et al. [6] find Epicopeiidae as sister to Uraniidae + Geometridae (Sematuridae were not included). In Mutanen et al. [9], Epicopeiidae group with Lasiocampidae, and Sematuridae is found as the sister-group of Uraniidae. Together Sematuridae and Uraniidae form the sister-group of Geometridae.

Minet [4] suggested the pointed and elongate ventral arm of the tegula was an apomorphy for Geometroidea (as defined in his article; Geometridae, Sematuridae and Uraniidae) and considered the presence of the sharp tegula in Epicopeiidae and Mimallonidae to be the result of parallel evolution. The ventral process of the tegula is indeed sharp in Sematuridae, Epicopeiidae, Geometridae, but also in Bombycoidea, Lasiocampoidea, Mimallonoidea and Noctuoidea, thus in most Macroheterocera. In Drepanidae (and most other Lepidoptera), the ventral arm of the tegula is blunt, (see Figures 48–53 in Minet [4]). The tegula of Uraniidae is elongate but spatulate. Minet and Scoble [83] suggested the most reliable autapomorphy to unite Geometroidea and Drepanoidea *sensu* Minet [4] (i.e. for Geometroidea the families Sematuridae, Uraniidae and Geometridae, and for Drepanoidea the families Epicopeiidae and Drepanidae) to be the strong anterior extension of pupal forelegs, and the presence of a transverse dorsal groove on pupal segment A10, yet with several secondary losses for both. The latter character was indeed found only among some representatives of these groups, while the former was highly homoplastic. Apart from these families, it was observed also in Bucculatricidae (Gracillarioidea), Attevidae and Praydidae (Yponomeutoidea), Urodoidea, Schreckensteinioidea, Pterophoroidea, Thyridoidea, Lasiocampoidea, Bombycoidea, Cimelioidea, most Papilionoidea and many Noctuoidea.

**Fig. 9:**
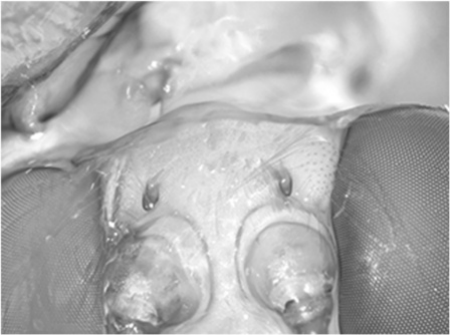
Protrusions behind antennae. *Sematura lunus*. Character is also present in *Urania leilus* and several geometrids (see Sihvonen & Kaila [86])

A morphological character in favor of the relatedness of sematurids with uraniids could be the knob behind the antennae, situated medially of the chaetosemata, present in both *Sematura* and *Urania* (Fig. 9). Similar structures, although with more variation in shape, from flat pores to larger triangular protrusions, were found widely in taxa belonging to Scopulini/Sterrhinae and singular representatives of Larentiinae and Archiearinae in a study by Sihvonen and Kaila [86]. Such protrusions could thus be found more widely in taxa belonging to Geometridae/Geometroidea.

Drepanoidea are monophyletic and associate with *Axia, Epicimelia* (Cimeliidae) and *Doa* (Doidae) (268: 77; 422: 80; 473: 82 %) (Figs. 2, 3, 4 and Additional files 8, 9, 12 and 13) as has also been suggested by recent molecular studies [6, 7, 9–12]. In several of the recent studies based on molecular data, Drepanidae are the sister-group of the other macroheteroceran superfamilies. In the analysis with the fastest evolving partitions removed and of the 268-taxon set, the clade comprising Drepanoidea, Cimeliidae and Doidae are sister to other macroheterocerans. However, when the partitions are not removed, the clade is nested in Macroheterocera (Figs. 2, 3 and 4). Minet and Scoble [83] proposed a modification of the larval mandibles, a large, flat area that is ventrally delimited by a carina, to unite Drepanidae and Epicopeiidae. The coding of this character was found to be continuous when other Lepidoptera, especially Macroheterocera, were examined in detail, and was thus impossible to code with any certainty, compromising its significance. It was excluded from the present analysis.

The sister-group relationship of Bombycoidea and Lasiocampoidea (473: 72 %) is recovered in the present result (Figs. 2, 3 and 4) as it has been in several studies based on genetic data [6–8, 10–12, 87]. In Mutanen et al. [9] and the ML analysis based on DNA-data only of the present study (Additional file 8), the sister-group relationship between the two superfamilies was not recovered suggesting that it is the addition of morphological data that brings about this relationship in the present result. In the reduced taxon sets (268 and 422), with less taxa from both superfamilies, the sister relationship is not recovered either. Instead, Lasiocampoidea are nested in Bombycoidea. In the ML tree of morphology only, Lasiocampoidea, although not monophyletic, are in a clade with Bombycoidea (Additional file 11). The adult character supporting the affinity of these superfamilies, also found in some other groups, is the closeness of the prescutal clefts in several taxa [4]. The taxon sampling for Bombycoidea in the present study is relatively low compared to the sampling of other large superfamilies. This is because the inter-familial relationships and splitting into subfamilies within Bombycoidea has been treated in more detail in recent phylogenetic studies [87, 88]. Brahmaeidae Swinhoe, 1892 and Lemoniidae Neumoegen & Dyar, 1894 were synonymized by Zwick [88] based on evidence from DNA and morphology. In the present result, *Brahmaea* does not show a close relationship to *Lemonia* and *Dactyloceras,* probably due to the lack of DNA and adult character data.

The monophyly of Noctuoidea is strongly supported (268: 100; 422: 99; 473: 93 %) and constituent families are also recovered as monophyletic in analyses based on combined (Figs. 2, 3 and 4) and DNA data (Additional file 8). In the analyses based on morphological data alone (Additional files 10 and 11), Noctuoidea are not monophyletic although they share the well-known synapomorphy, i.e. the metathoracic tympanal organs. The other established synapomorphy, the presence of two MD setae on larval metathorax [89], was excluded in the present analysis due to the impossibility to code it because many of ‘macrolepidopteran’ larvae are covered with microsetae that hide the possible presence of this character in this assemblage. The monophyly of Noctuoidea has convincingly been recovered in all recent molecular studies, and its family systematics with both morphological and molecular insight, has been elucidated by Zahiri [31, 90–92]. Therefore, we do not discuss this superfamily further here.

## Discussion

Our objective was to rigorously explore the morphology of ditrysian Lepidoptera and to analyze the extensively coded data to infer a hypothesis for the phylogenetic relationships within Ditrysia. The aim was to obtain supplementary insight from morphology to that derived from DNA-based data, and compare this information to the recently published DNA-based phylogenetic hypotheses [6–12]. We consider this a way to supplement, evaluate and weigh these partially conflicting hypotheses. A feature in common to all these DNA-based studies is the weak or non-existing support for groups among the most basal and the most “derived” taxa, i.e. for the so-called ‘backbone’ of the lepidopteran phylogeny. For this reason, we put special emphasis on acquiring as comprehensive data as possible on the groups that supposedly belong to this area of lepidopteran phylogeny. Somewhat lesser attention was paid to inter-family relationships within superfamilies that have recently received specific attention [25, 31, 55, 59, 60, 87, 93, 94]. We combined morphological data obtained from larvae, pupae and adult specimens, coded from nearly the same sampling of exemplar taxa as in the study by Mutanen et al. [9]. The sampling covers nearly all recognized ditrysian families with a comprehensive representation of their subfamilies, and is supplemented with a number of taxa with unclear affinities.

As has been the result in DNA-based studies, even comprehensive morphological data were not able to bring forth strong support for any particular hypothesis on the interrelationships of the backbone Ditrysia. Possible reasons for this are the amount of homoplasy, general lack of informative characters except for apical nodes, conflicts between morphological and molecular data, and our decision to include and retain unstable taxa in the analyses. These matters are discussed below.

### Lack of informative characters

Problems in inferring deeper level relationships in Ditrysia with molecular methods have been explained by ancient rapid radiation of most major lepidopteran lineages (e.g. [6]). In ancient rapid radiation, lineage splitting occurs with close temporal spacing and very few character patterns are left as evidence of the relatedness of lineages that diverged from one another. The little phylogenetic signal that remains may be obscured by biases in sequence data (heterogeneity in nucleotide and amino acid composition, unequal rates of evolution across sites or lineages, non-independent substitutions and selection), and/or by homoplastic changes on the apical branches [95]. Rapid radiation and difficulties in inferring relationships among lineages united by short nodes is a problem in phylogenetic analyses of many insect groups that have undergone spectacular diversification in a short period of time in the Cretaceous-Tertiary [18]. Recent divergence time estimates also suggest a significant increase in the diversification rate in Ditrysia around this time period, 90 million years ago [96]. Our examination of the morphology across the ditrysian superfamilies and families also confirms the lack of clear morphological characters for making inferences about these deeper evolutionary relationships. A possible explanation for this is that the first taxa of each monophylum supposedly had, and their extant progeny may still retain, predominantly plesiomorphic traits compared to the more derived members of the clade. Paucity of apomorphies among the basal representatives thus leads a predictable pattern that evidence for inferring interrelationships of monophyla is scarce, if such ‘ancient’-looking taxa still exist and are included among the studied taxa.

The collected morphological data were found useful in reconstructing evolutionary relationships at the apical nodes. Our study affirms that most superfamilies are diagnosed by at least one fairly clear apomorphy, although diagnostic characters may not be found in all life stages. However, the morphology-only analyses were unable to recover some of the well-established superfamilies. An intriguing feature in our results is that even though no clear synapomorphies have been proposed to diagnose certain superfamilies nor were such characters found in the course of the present study or those suggested in earlier studies were refuted, such superfamilies were nevertheless also recovered in morphology-only analyses. An example is the superfamily Gracillarioidea.

Although the exemplar taxa in the present study represent most of the ditrysian superfamilies, it has to be kept in mind that an unknown number of lineages have likely gone extinct in the millions of years that have followed the advent of Ditrysia. It is possible that along with the extinction of these lineages informative characters (morphological and molecular) have disappeared making the estimation of phylogenetic relationships among the ditrysian superfamilies even more difficult, just as in cases of insufficient taxon sampling where important phylogenetic information is missing. At present, it is not known how heavily, for example, the Cretaceous-Paleogene mass extinction event affected the diversity of Lepidoptera [96].

The observed morphological homogeneity of Ditrysia at the deeper level can safely be considered to be due to real biological and historical causes, but limitations on character coding are also set by methods used or material available. Some characters of potentially great influence may have been coded incorrectly for some taxa due to difficulties in making reliable observations. Many such characters were probably overlooked, and others had to be excluded from the final analysis. Examples include the presence or absence of the pupal frontoclypeal and epicranial sulci, which are easier to observe in exuviae than in pupae with uneclosed adults. Many setal characters of larvae were excluded from the majority of the Macrolepidoptera, as their final-instar larvae are usually covered by small setae that obscure the pattern of primary setae. This problem could have possibly been overcome if first-instar larvae could have been examined instead of final-instar larvae. This would have, however, dramatically reduced sampling due to the lack of available material. Another option, using final instar larvae, supplemented with first instar larvae, would have been potentially misleading, as it would have been impossible to avoid duplication of some features in the data because of problems of homology.

A problem in coding adult characters is the high plasticity of structures and thus the difficulty of dividing characters into discrete states. Many characters are easily delimited when only a subset of the taxa is examined, i.e. only extreme states are observed, but the boundaries become exceedingly blurred as more taxa are examined and more variation and intermediate forms are encountered. Many characters had to be discarded from the final analyses because of uncertain or artificial boundaries of states. Typically, e.g., the degree of sclerotization in various body parts, and variation in strength of sutures and sulci, proved impossible to code unambiguously across the taxa. The use of morphometric tools could arguably increase the amount of character data as continuous characters such as length, size and details of shape could possibly be objectively divided into character states. Uncertainty over the homology of certain structures (e.g. of lamellae and extensions of the metathoracic furca) across all groups was also a problem. Certain structures may be related to the size of the insect, but these are not easy to detect and interpret. This was also a reason for our decision to omit wing venation and male genital characters entirely.

Both morphological and molecular characters contain information about the phylogeny of organisms but the importance of morphological data in the future of phylogenetics has been disputed see e.g. [97–99]. The problem with morphological data is less with quality than with quantity. Giribet [100] discusses how morphology could be made “scalable” by utilizing new techniques that allow larger acquisition of morphological and anatomical data. Such new techniques include immunostaining and confocal laser microscopy, and also 3-D imaging techniques that allow reconstructions of the internal structures of organism without having to destroy them [101–103]. This enables the non-destructive study of rare museum specimens, ultrastructure of insects in amber fossils [104] and in some cases live specimens [105]. Other new techniques to add to the list are magnetic resonance imaging (MRI) [106], advanced digital microscopy, computer-assisted tomography and image recognition [107, 108]. The tools to share and work on morphological information have also undergone a revolution, e.g. Morphbank : : Biological Imaging (http://www.morphbank.net/), MorphoBank [33] and various discussion groups and web forums (e.g. The Lepidoptera dissection Group, http://www.dissectiongroup.co.uk/).

### Homoplasy

The frequency of homoplasy (i.e. independently evolved or reduced characters) was found to be high in both larval and pupal characters. Therefore, few characters of the immature stages, according to our analysis, are useful in diagnosing monophyletic groups. Not even the non-ditrysian vs. ditrysian grades could be distinguished by data coded from immature stage morphology alone. Also, the presumably strong pupal structures that according to previous hypotheses [3, 4] distinguish basal groups of Ditrysia and Obtectomera failed to recover these taxa unequivocally. One possible explanation is the repeated shifts to different modes of life in Ditrysia, as has been shown to be the pattern at lower phylogenetic levels too [30].

Extreme examples of taxa with significant amounts of convergence are leaf-mining larvae. These larvae often have considerable convergent modifications in their morphology and reduction of structures. Parsimony analyses of morphology may fail to group taxa correctly if the level of homoplasy is very high [109]. Examples of such taxa in our data are Lyonetiidae, Douglasiidae, *Ochsenheimeria* and *Millieria.* Another case of obvious convergence is the tendency of diurnal lepidopterans in a number of clades to have clubbed antennae (e.g. in Castniidae, Zygaenidae, Thyrididae, Sematuridae, Noctuidae (Eucocytiinae) and Papilionoidea. These examples demonstrate the potential effect of convergence on the overall topology, especially when there are several traits that by adaptation have led to appearance similar enough to make their recognition as distinct features hard. In many cases more thorough study of the morphology of allegedly homologous characters, e.g. “clubbed” antennae, is needed as new information could reveal them to be quite distinct despite their superficial similarity.

A morphological trait in Lepidoptera regarded to be phylogenetically very significant is the larval proleg shape. It has often been used as a distinguishing feature to separate ‘Macrolepidoptera’ from ‘Microlepidoptera’, but appears to be convergent in several lineages, viz. Schreckensteinioidea – Urodoidea, Zygaenidae, Papilionoidea and Macroheterocera, but excluding Pyraloidea. These groups usually have the following basic structure of the proleg: the base of the proleg is elongate and forms the greater part of the proleg; the proleg planta is asymmetric, laterally bulbous, and crochets are usually arranged as longitudinal mesoseries. The usual ‘microlepidopteran’ type of proleg consists of a small proleg base that is little more than a ring encircling the planta. The planta is either reduced or cylindrical, but not asymmetric, and the crochets most often are formed as a full or nearly full circle. Even though different combinations of these features were observed in some taxa, the “macrolepidopteran” combination is similar in Papilionoidea s. lato and Macroheterocera. These groups have, as a rule, a similar larval mode of life as they are external leaf-feeders, usually gnawing on the edge of the leaf of host plants. This favors adaptations of the proleg shape to optimize the grip on the edge of the leaf. Most pyraloid larvae live more or less concealed and do not need such specialized proleg modifications. The structure of larval prolegs may play a crucial role in the topology obtained in analyses with morphological data, as the shape is not one feature, but a combination of several traits. When a set of such non-independently evolved characters are coded from non-related taxa, they may have enough weight to pull the taxa together.

The high level of homoplasy in the morphological traits is in itself interesting. Just as DNA can evolve fast enough to reach phylogenetic saturation, it has been shown that morphological characters can also show saturation [110]. Constraints exerted by intrinsic causes such as development, function or persistent selective trends do not allow for the continuous rise of new characters. Clades seem to exhaust their available character space and this leads to homoplasy. However, convergence does not only concern the morphology of organisms. Genes can also be under selective pressure for a particular function and converge at the nucleotide level [111, 112].

Although we aimed to avoid size-related characters in our morphological matrix, and tested the effect of removing characters we suspected could be correlated with size (e.g. distance between prescutal clefts on mesothorax), characters or character combinations less obviously correlated with size may have remained. As the signal for the deep nodes is weak, it is possible that such characters led to artificial clustering of groups by species’ overall size.

### Combining and comparing morphological and molecular evidence

Even though morphological characters comprised only 8 to 10 % of the total amount of data, depending on the analysis, the incorporation of morphological data had, in places, significant effects on the results. Adding morphological data established more stable positions for some taxa, such as Cyclotornidae and Epipyropidae, which in molecular analyses have defied well-established positioning within their putative superfamily. Likewise, the monophyly of Sesioidea (without Choreutidae) and Cossoidea were now recovered, even though with entirely different morphological traits to those suggested earlier. Tineoidea excluding *Metapherna* were recovered as monophyletic. In DNA-based studies *Metapherna,* an alleged tineid not included in previous analyses, was never found to associate with Tineoidea. Rather, it was recovered to form its own clade linking Tineoidea and the more advanced Lepidoptera – a result already hinted at by Robinson and Nielsen [58].

It is not uncommon that morphology and genes tell different stories about the evolutionary history of organisms. This is caused by the differences in the rates of change and the evolutionary mechanisms acting on these rates. Conflict between datasets and resulting phylogenies may be caused by convergent evolution, methodological reasons (missing data, polymorphism, continuous variation, incorrect or inaccurate evolutionary models), or uncertain homology assessment. Cases of conflict between datasets are intriguing as they may allow the identification of the underlying biological processes causing the conflict [112].

When conflicting clades arise in analyses, it is important to consider the effect of the methods chosen to analyze the data, but also to seek alternative evidence and information about the ecology of the organisms. Combined with the molecular dataset consisting of eight gene regions, the morphological data were able to distort not only weakly supported relationships, but also fairly strongly supported ones, such as the interrelationships in Papilionoidea [9, 10]. Without alternative sources of evidence, conflicting results would be difficult to evaluate. Recent studies based on different sets of genes to those in the present study provide some insight into the matter. The taxon sampling of those studies has been, however, often lower and not permitting simple comparison. Also, the high support values for proposed relationships in low taxon sampling studies based on transcriptomic, genomic or proteomic data may be misleading. In the absence of more closely related taxa, more distantly related taxa may be drawn together but still obtain relatively high support values [19]. This phenomenon is illuminated by the conflicting hypotheses with strong support values in large molecular data sets of (e.g. [6, 8]). On the other hand, many patterns with low support observed in the study of Mutanen et al. [9] with dense taxon sampling, but lower amount of molecular data, have subsequently been obtained in other studies with much larger gene sampling.

Conflicting patterns of relationship between molecular and morphological data, each with good support, is found, for example, in the position of Douglasiidae. The morphological result always places this family in Gracillarioidea, as in the superfamily concept of Davis and Robinson [61], also based on morphology. However, molecular evidence is strongly in conflict with this result, unequivocally placing them in Apoditrysia, which is also the case for our combined data analysis.

### Unstable taxa

In this study, we attempted to cover the diversity of ditrysian Lepidoptera as comprehensively as possible. Therefore, we paid special attention to the inclusion of taxa with unknown phylogenetic positions, or which have been unstable in recent studies based on DNA-data. Our aim was to examine whether the combination of extensive morphological data could provide information to infer their likely relationships. We presumed that taxa appearing unstable in genomic data are not necessarily the same as those appearing as such in morphological data. We further expected that in analyses with both types of data combined at least some of the rogues would become more stable.

Combined data indeed gave moderate support at least in some analyses (268: 79; 422: 76; 473: 60 %) for the inclusion of Epipyropidae and Cyclotornidae in Zygaenoidea, a result that in molecular studies had either not been obtained, or only with a weak indication for Epipyropidae. The combination of data also brought out the sister-group relationship between Bombycoidea and Lasiocampoidea, not previously recovered with DNA data similar to those used in this study. Although bootstrap support is not strong (50–56 % in the combined analyses), the evidence supporting the monophyly of Schreckensteinioidea + Urodoidea seems a plausible hypothesis when morphological data are also considered. Similarly, when compared with results of studies based on molecular data only, the association of Galacticoidea with Tortricoidea also gains moderate to strong support in our analyses (268: 81; 422: 52; 473: 44 %) for the first time. A monophylum comprising Alucitoidea, Epermenioidea and Carposinoidea is repeatedly recovered in the analyses of combined data, although with low support values. Adding morphological information also increased support for the placement of Prodidactidae in Hyblaeoidea. On the other hand, there are cases where sequence data contradicts morphological information. For instance, genetic data strongly suggests that Douglasiidae are not gracillarioids, but apoditrysians.

Nevertheless, combining data did not stabilize the position of many taxa that have been unstable in previous studies. The unassigned *Heliocosma* group, Choreutidae and Immoidea are typical examples of such taxa. As the “backbone” of ditrysian Lepidoptera did not gain much further support from combining morphology and molecular data, one can argue that several of the larger assemblages behave in an unstable manner, with presumably close groups displaying alternative arrangements among each other, e.g. Calliduloidea, Papilionoidea and Thyridoidea. The Cossoidea + Sesioidea + Zygaenoidea assemblage is particularly intriguing as it displays different positions and compositions among data sets. In the results of morphology-only analyses, Zygaenoidea were not unambiguously associated with Cossoidea + Sesioidea, which, in turn, often form a sister-group relationship with tineoid group families. In the combined data with all third codon positions included, this three-superfamily assemblage was placed within Obtectomera. This relationship was broken when the third codon position nucleotides were removed.

An intriguing pattern in our data is that several of the unstable taxa are internal feeders. These include Cemiostominae, Lyonetiinae, Bedelliidae, Heliodinidae (all Yponomeutoidea), Millieriidae and Douglasiidae. Similar trends have been found in Gelechioidea, where a wide array of life habits is found [25, 30]. Although not tested using quantitative methods, these taxa are often observed to display disproportionally long branches and more often display multiple positions in phylograms. This raises the question whether shifting to leaf-mining or other demanding life modes requires not only considerable morphological adaptation, but also rapid genetic evolution in many loci. This would support the notion that taxa with long branches, i.e. those with a disproportionally fast evolutionary rate, would have a higher likelihood to behave as rogues. In such cases, adding more genetic data might not be able to solve the problem. Another example of taxa with an unusual mode of life are Epipyropidae and Cyclotornidae, whose larvae have a two-phased development, and are ectoparasites of other insects; a life-history trait that is possibly associated with rapid molecular evolution [69]. As discussed above, stable placement of these families has not been reached with DNA data.

## Conclusions

Our results support previous assertions of the paucity of clear morphological characters diagnosing larger assemblages of ditrysian superfamilies. Previously proposed characters are tenable to some extent but alone do not allow the unequivocal phylogenetic placement of taxa. The degree of homoplasy is high and likely hid phylogenetic signal in the analyses. Nevertheless, most currently recognized superfamilies were recovered, and were found to have at least one previously recorded diagnostic character. In some cases, the suggested apomorphies appeared to be supported only weakly, or remained entirely unsupported (e.g. Thyridoidea).

Examination of the distribution of over 500 morphological traits across Ditrysia revealed characters bearing evidence on the evolutionary relatedness among smaller groups, such as families (Alucitidae nested within Tineodidae) and in some cases superfamilies (e.g. Schreckensteinioidea + Urodoidea, Yponomeutoidea + Gracillarioidea). These characters also have potential use in identification keys and in identification of fossil samples. The inclusion of morphological data also allowed to “pin down” several taxa that for some reason appear unstable in analyses of molecular data.

In the near future, we will begin to see the results of the first large-scale studies based on genome-wide data aimed at solving the deep divergences in the evolutionary tree of Ditrysia. The morphological data collected in the course of this study will certainly prove useful in evaluating and validating the results. Likewise, the results of future phylogenomic studies will help explain the distribution of some of the morphological characters and identify possible misinterpretations.

Comparing structures across the ditrysian subfamilies will also gain us insight into the evolution and diversity of morphological characters. The distribution of these characters and their reversals across the different clades can be used in future studies on the evolution of these traits.

### Availability of supporting data

The data sets supporting the results of this article are available as Additional files 1, 2, 3, 4, 5, 6, 7, 8, 9, 10, 11, 12 and 13. Sequence data are available in GenBank (Accession numbers in Additional file 4). Morphological character data are available in Morphobank, Project 2183, http://www.morphobank.org/.

## Additional files

Additional file 1:
**List of exemplar taxa in study.** (XLS 224 kb) Additional file 2:
**Sequence lengths.** (XLSX 48 kb) Additional file 3:
**Gene summary.** (XLSX 10 kb) Additional file 4:
**GenBank accession numbers.** (XLSX 57 kb) Additional file 5:
**Character list.** (DOCX 49 kb) Additional file 6:
**Character matrix.** (TXT 218 kb) Additional file 7:
**Phylogenetic tree from maximum likelihood analysis of combined data with third codon position removed from molecular data except from EF1a.** 473 taxa, 4991 characters (4461 bp, 530 morphological characters). (PDF 35 kb) Additional file 8:
**Phylogenetic tree from maximum likelihood analysis of DNA data with third codon position retained, 422 taxa, 6172 bp.** (PDF 32 kb) Additional file 9:
**Phylogenetic tree from maximum likelihood analysis of combined morphological and molecular data, 473 taxa.** Molecular data were categorized according to the rate of evolution with the program Tiger and partitioned with RatePartitions. Two fastest evolving partitions (partition 7 with 482 bp and partition 6 with 357 bp) removed. (PDF 33 kb) Additional file 10:
**One of the 19 equally most parsimonious trees (length = 5305 steps, Ci 12, Ri 62) from the parsimony analysis of morphological data, 318 taxa, 530 characters.** Additional file 1 lists morphological data for 320 taxa, but in the present analysis, data for two species of *Thereutis* were concatenated into one terminal taxon as were those of two species of *Cyclotorna* making the total number of taxa in the analysis 318. (PDF 4061 kb) Additional file 11:
**Phylogenetic tree from maximum likelihood analysis of morphology, 318 taxa, 530 characters.** Additional file 1 lists morphological data for 320 taxa, but in the present analysis, data for two species of *Thereutis* were concatenated into one terminal taxon as were those of two species of *Cyclotorna* making the total number of taxa 318. (PDF 21 kb) Additional file 12:
**Phylogenetic tree from maximum likelihood analysis of combined morphological and molecular data, 268 taxa.** The data set includes all the taxa for which both morphological and molecular data were available. (PDF 18 kb) Additional file 13:
**Phylogenetic tree from maximum likelihood analysis of combined morphological and molecular data, 422 taxa.** The data set includes all the taxa for which DNA data were available. Morphological data were available for 268 of these taxa. (PDF 28 kb)
