# Supplementary material for: Elusive ditrysian phylogeny: an account of combining systematized morphology with molecular data (Lepidoptera)
Source: BMC Evol Biol. 2015 Nov 21;15:260. doi: 10.1186/s12862-015-0520-0 (PMC4654798; doi:10.1186/s12862-015-0520-0)
Supplement: Additional file 5: — Character list. (DOCX 49 kb) [file 12862_2015_520_MOESM5_ESM.docx]

Appendix 4: **CHARACTER LIST**

Characters 82, 90, 197, 248 and 249 were not included in analyses because they are uninformative with the taxon sampling of the present study.

The terminology of larval characters follows Hinton (1946) with conventions adopted by Stehr (1987), and that of pupae Patočka & Turčani (2005).

Adult characters follow mostly the terminology of Scoble (1992) and Kristensen (2003). See also Data matrix in Mophobank (project 2183) for references and notes on adult morphology.

More references at the end of the list.

**LARVAL CHARACTERS**

1. Larval diet: on green plants (0); on dead material or fungi (1); ectoparasite on other insects (2).

2. Hypermetamorphosis with sap feeding first instars and variably modified last instar stage: absent (0); present (1).

3. Hypermetamorphosis with disproportionately large head and thorax in first instar: absent (0); present (1).

4. Presence of a hibernaculum i.e. a spun cocoon for moulting or a quiescent larval instar: absent (0); present (1).

5. Larva with a portable sack: no (0); yes (1).

6. Shape of larval head: halves of head capsule convex making the head more or less rounded (0); halves of head capsule flat so the whole head is flat (1).

7. Caudal part of larval head: halves of head capsule evenly convex (0); halves of head capsule projected caudally to make the head somewhat bifid (1); halves of head capsule dorsally tapered to form nearly or entirely pointed apex (2).

8. Posture of larval head: hypognathous (0); semiproganthous (1); prognathous (2).

9. Larval head: not retractile into thorax (0); retractile into thorax (1).

10. Triangular cap dorsally covering larval antenna: absent (0); present (1).

11. Rounded cover over larval antenna: absent (0); present (1).

12. Ventral part of head capsule separated by furrow or a branch of ecdysial line between antenna and clypeus at the base of mandible: no (0); yes (1).

13. Secondary setae on head: absent (0); present arising from non-erected pinacula (1); present arising from strongly erected pinacula setae basally conspicuously thickened (2).

14. Dense coverage of integumental hairs on head: absent (0); present (1).

15. Reticulate sculpture in head: absent (0); present (1).

16. Rugous sculpture consisting of minute pits on head: absent (0); present evenly covering head (1); present in groups forming intermittent reticulate smooth areas (2).

17. Head porose i.e. with deep pits: no (0); yes (1).

18. Adfrontal suture: ecdysial line in front of antenna (0); behind antenna (1).

19. Ecdysial line of adfrontalia: reaches cranial incision (0); does not reach cranial incision (1).

20. Epicranial suture: present at least in adfrontal area (0); entirely absent (1).

21. Transverse furrows on frons: absent (0); present (1).

22. Longitudinal furrows on frons: absent (0); present (1).

23. Depression on frons: absent (0); present (1).

24. Depression or pit along anterior ventral part of margin of frons often most pronounced at base of seta F1: absent (0); present (1).

25. Entire margin of frons depressed: no (0); yes (1).

26. Posterior dorsal end of frons in pit: no (0); yes (1).

27. Width of anteclypeus: narrow at most 1/3 of the width of labrum (0); about half the width of labrum (1); almost or as broad as labrum (2).

28. Notch of lower margin of labrum: deeper than breadth of labrum in ventrocaudal direction (0); distinctly less deep than breadth of labrum in ventrocaudal direction (1); nearly or entirely absent i.e. lower margin of labrum almost straight (2).

29. P setae of head: two (0); one (1); none (2).

30. Stemmata 1-4: separate from each other (0); approximate to each other (1); stemmata in a stalked raspberry like cluster (2).

31. Presence of stemmata 1 and 2: both present (0); one or both absent (1).

32. Stemmata 1 and 2: not approximate to each other compared to other stemmata (0); approximate to each other (1).

33. Stemmata 3 and/or 4: absent not possible to identify which (0); present (1).

34. Size of stemma 3 sometimes also 4: about the same size as other stemmata (0); disproportionately large as compared to other stemmata (1).

35. Stalking of stemmata: absent (0); at least some stemmata stalked most often stemmata 2-5 (1).

36. Stemma 5: not separated from other stemmata (0); separated from other stemmata (1).

37. Stemma 6: absent (0); present (1).

38. Position of stemma 6: about in the middle of the stemmatal ring (0); distinctly lower than the middle of the stemmatal ring (1).

39. Primary setae of mentum: widely apart of each other (0); approximate (1); absent (2).

40. Secondary setae on mentum: absent (0); present (1).

41. Sculpture of mentum: smooth (0); dentate (1); granulose (2); with lens shaped sculpture (3).

42. Eversible bilobed structure (possibly a gland) between pre and submentum: absent (0); present (1).

43. Tongue shaped lobe laterad of submentum: absent (0); present (1).

44. Oval pit on submentum of larval head: absent (0); present (1).

45. Sclerotized pair of grooves on submentum of larval head: absent (0); present (1).

46. Length of basal segment of larval labial palpus: at most 1.5 times its diameter (0); at least twice its diameter (1).

47. Length of second segment of larval labial palpus: at most 2 times as long as its diameter (0); over three times as long as its diameter (1).

48. Position of larval labial palpus: laterad of spinneret (0); dorsolaterad of spinneret (1); approximately dorsad of spinneret (2).

49. Larval labial palpus: tubular (0); with mesal lobe (1).

50. Shape of fusuliger: triangular dorsally situated (0); short and broad (1); tubular (2).

51. Length of the fusuliger: very short shorter than its width (0); 1-2 times its width (1); at least 3 times longer than wide (2).

52. Shape of fusulus: straight (0); upward bent (1); downward bent (2).

53. Transsection of fusulus: more or less rounded (0); flat (1); very broad and narrow (2).

54. Shape of apical part of fusulus in dorsal or dorsoanterior view: parallel sided (0); tapered as hair like (1).

55. Distal lobes on spinneret: absent (0); present (1).

56. Size of stipular setae: minute or absent (0); well discernible but distinctly shorter than premental setae (1); almost or quite as large as premental setae (2).

57. Distinctive neck between head capsule and thorax: absent (0); present (1).

58. Eversible ventral gland adenosma between head and prothorax: absent (0); present (1).

59. Shape of adenosma between head and thorax: single ventral pouch (0); paired ventral pouch (1); pair of dorsolateral swellings (2).

60. Eversible lateral gland on larval prothorax: absent (0); present (1).

61. Eversible bifurcate dorsal gland osmeterium on larval prothorax: absent (0); present (1).

62. Lateral sclerotised ridgeshaped armature: absent (0); present (1).

63. Conspicuous scales that laterally surround whole body laterally associated with L setae: absent (0); present (1).

64. Discernible primary setae on last instar larva. If larva is covered by secondary setae this character is coded as present (0); absent (1).

65. Shape of primary setae: setiform (0); plumose (1); distally clear cut or branched to form triangular fan shaped apex (2); club shaped (3); stout erected from star shaped pinaculae (4).

66. Pinacula of SD and L setae: indistinct or if distinctly sclerotised not erected (0); erected (1).

67. Pinacula of D setae: indistinct or if distinctively sclerotised not erected (0); erected (1).

68. Secondary setae arranged as verrucae i.e. dense seta groups situated usually at or around the places of primary setae: absent (0); present (1).

69. Secondary setae scattered over the cuticle outside prolegs: absent (0); present (1).

70. Shape of secondary setae: setiform (0); spatulate (1); stout erected from star shaped pinacula (2).

71. Melanised erected pinaculae at the base of secondary setae or at their place if they are not developed: absent (0); present (1).

72. Sculpture on cuticle: absent (0); present (1).

73. Cuticular sculpture shaped as microtrichia scattered over the cuticle: absent (0); present (1).

74. Cuticular sculpture shaped as star or triangular-shaped over the cuticle: absent (0); present (1).

75. Cuticular sculpture lens-shaped: absent (0); present (1).

76. Larval spiracle of T1: rounded or oval, vertically positioned (0); oval, horizontally positioned (1).

77. At the place of spiracle in T2-3: nothing (0); like a remnant of external spiracle (1).

78. Ventral sclerotization on larval prothorax: absent (0); present (1).

79. Dorsal shield of larval prothorax: not extended laterally to include L setae or spiracle (0); extended laterally to include L setae (1); extended laterally to include L setae and spiracle (2).

80. Sclerotised shield on larval mesothorax dorsally: absent (0); present (1).

81. Sclerotised shield in larval metathorax dorsally: absent (0); present (1).

82. Dorsal seta group of thoracic segments T2-3: absent (0); present (1).

83. Approximation of dorsal setae on thoracic segments T2 3 D1 and D2 approximately on same level their pinacula often fused (0); D1 much cephalad of D2 (1).

84. Length of D1 of thoracic segments T2-3: about as long as D2 (0); distinctly shorter than D2 (1); longer than D2 (2).

85. L group of prothorax: trisetose (0); bisetose (1); unisetose (2); with no setae present (3).

86. L group of T2-3: trisetose (0); bisetose (1); unisetose (2).

87. SV group of meso and metathorax unisetose (0); bisetose (1); trisetose (2); quadrisetose (3).

88. Thoracic legs: entirely developed (0); absent or at most coxa discernible (1).

89. Thoracic coxae: arising from integument (0); on erected often more or less sclerotised base (1).

90. Femur of thoracic leg: not distally swollen (0); distally swollen (1).

91. Claw of thoracic leg: absent (0); present (1).

92. Axial seta on claw of thoracic leg: absent (0); present (1).

93. Scaly or trichiose coverage on tarsi of thoracic legs: absent (0); present (1).

94. A hook distoventrally at thoracic tarsi: absent (0); present (1).

95. Shape of claw of thoracic legs: curved with rounded or to varying extent conical ventral broadening basal loop (0); elongate (1).

96. Tarsal seta 1 of thoracic legs: not differentiated from other setae on any thoracic legs (0); thicker than other setae on at least some thoracic legs or clavate or fan shaped there are intermediates between these types therefore they are considered as one character (1); hair shaped thinner than other thoracic setae on at least some thoracic legs (2).

97. Tarsal seta 2 of thoracic legs: not differentiated from other setae on any thoracic legs (0); thicker than other setae on at least some thoracic legs or clavate or fan shaped (1); hair shaped thinner than other setae on at least some thoracic legs (2).

98. Tarsal seta 3 of thoracic legs: not differentiated from other tarsal setae on any thoracic legs (0); thicker than other tarsal setae at least on some thoracic legs or clavate or fan shaped (1); sucker shaped (2).

99. Tarsal seta 4 of thoracic legs: not differentiated from other tarsal setae on any thoracic legs (0); thicker than other setae on at least some thoracic legs or clavate or fan shaped (1).

100. Fusion of prothoracic coxae: separate may be approximate (0); fused (1).

101. Fusion of mesothoracic coxae: separate may be approximate (0); fused (1).

102. Fusion of metathoracic coxae: separate may be approximate (0); fused (1).

103. V setae on T1: absent (0); present (1).

104. V setae on T2-3 absent (0); present (1).

105. Position of V setae of prothorax: at mid-point between coxae of thoracic legs (0); caudad of mid-point between coxae of thoracic legs (1).

106. Position of V setae of meso- and metathorax: at mid-point between coxae of thoracic legs (0); caudad of mid-point between coxae of thoracic legs (1).

107. Shape of metathoracic leg: not different from other thoracic legs (0); pistol shaped (1).

108. Erect distally widened fungus shaped protuberances laterally in A1-3: absent (0); present (1).

109. Oblique gland in A1 within L group of setae: absent (0); present (1).

110. Size of spiracle of A8: larger than those of A1 7 (0); of equal size o those of A1 7 (1); enormously enlarged (2).

111. Position of spiracle of A8: about as level with other spiracles (0); distinctly more dorsally positioned than other spiracles (1).

112. Spiracles: not stalked (0); stalked (1).

113. All D setae of A1 8 in common pinaculum: no (0); yes (1).

114. Dorsal seta D1 of abdomen: absent (0); present (1).

115. Length of dorsal seta D1 of abdominal segments A1-8: as long as D2 (0); shorter than D2 (1); longer than D2 (2).

116. Position of dorsal seta D1 of abdominal segments A1-8: dorsad of or at level with D2 (0); laterad of D2 (1); approximate in same pinaculum (2).

117. Position of dorsal seta D2 of A1-8: separate from SD1 (0); in the same pinaculum as SD1 (1).

118. Length of subdorsal seta SD2 of abdominal segments A1-8: as long as SD1 (0); well-developed but shorter than SD1 (1); minute or absent (2).

119. Seta L1 of A1-8: absent (0); present (1).

120. Setae L1 and L2 of A1-8: setae approximate, often in same pinaculum (0); setae distant, in their own pinacula (1).

121. Presence of paired ventral appendage, may be wart or sucker without crochets on A1 at least at some life stage: absent (0); present (1).

122. Presence of paired ventral appendage, may be wart or sucker without crochets in A2 at least at some life stage: absent (0); present (1).

123. Presence of proleg on A3: absent (0); present (1).

124. Presence of proleg on A4: absent (0); present (1).

125. Presence of proleg on A5: absent (0); present (1).

126. Presence of proleg on A6: absent (0); present (1).

127. Presence of ventral paired appendage, may be wart or sucker on A7 at least at some life stage: absent (0); present (1).

128. Presence of ventral paired appendage, may be warts or suckers on A8 at least at some life stage: absent (0); present (1).

129. Prolegs of A3-6: arise from integument (0); arise from erected base making the pairs look basally somewhat fused (1).

130. Fusion of prolegs of A3-6: prolegs on each segment separate (0); fused mesially (1).

131. Distance between prolegs on A3-6: all prolegs about similar distance from each other (0); prolegs on A 4-5 approximate (1).

132. Shape of proleg base: little more than a ring circling the planta (0); elongate and forms the greater part of the proleg (1).

133. Shape of planta: either reduced so that the crochets appear to arise directly from the venter or cylindrical symmetric (0); bulbous laterally asymmetric (1); cylindrical gradually widening towards apex (2).

134. Lateral sclerotised reinforcement in the middle of planta: absent (0); present (1).

135. Median narrowing on prolegs: absent (0); present (1).

136. Crochets on prolegs of A 3-6 when proleg present: absent (0); present (1).

137. Seriality of crochets in prolegs of A3-6: multiserial (0); uniserial (1); biserial (2).

138. Arrangement of crochets on prolegs of A3-6: full or nearly full circle (0); lateral penellipse (1); mesal penellipse (2); pair of transverse rows (3); longitudinally arranged mesoseries (4).

139. Crochets on prolegs of A3-6: homoideus (0); heteroideus (1).

140. Separate lateroseries of crochets: absent (0); present (1).

141. Irregular area of undifferentiated spines in prolegs: absent (0); present (1).

142. SV group of A1-8: absent (0); present (1).

143. SV group of A3-6: on their own swelling appearing separate from proleg base (0); on proleg base (1).

144. Number of setae in SV group on prolegs of A3-6 last instar larva: one (0); two (1); three (2); four to six (3); numerous (4); State 5 (5).

145. V group on A1-8: absent (0); unisetose (1); bisetose (2).

146. Secondary setae on prolegs: absent (0); secondary setae scattered over the cuticle of anal prolegs present (1); secondary setae at least some other prolegs present (2); State 3 (3).

147. Approximation of prolegs on A10: separate (0); closely set not arising from a common protuberance (1); closely set arising from a common protuberance (2).

148. Crochets on proleg of A10: absent (0); present (1).

149. Arrangement of crochets on prolegs of A10: full or nearly full circle (0); transverse often curved row may be more to lateral or mesal side extended (1); mesoseries (2).

150. Crochets on prolegs of A10: homoideus (0); heteroideus (1).

151. Ring-shaped pinaculum around SD1 leaving a non sclerotised area around the seta at A1-8: absent (0); present (1).

152. Middorsal eversible gland at A6 and A7: absent (0); present (1).

153. Middorsal pads with deciduous short setae: absent (0); present at A1 9 (1); present in all body segments (2).

154. Triangular swelling on A8 dorsally: absent (0); present (1).

155. Horn-shaped lobe mid-dorsally on A8: absent (0); present (1).

156. Reduction of larval segments A8 and A9: not reduced (0); reduced (1).

157. Shape of SD1 on A9: as other setae (0); hair like (1).

158. Arrangement of D1 and D2 on A9: D1 ventrad of D2 (0); D1 dorsad of D2 (1); both at the same pinaculum (2).

159. Size of D1 on A9: approximately as D2 (0); distinctly smaller than D2 (1); absent (2).

160. Position of dorsal seta D1 of A9 in relation to SD1: separate from SD1 (0); in the same pinaculum as SD1 (1).

161. Approximation of D2 setae of A9: D2 setae separate from each other (0); D2 setae in common pinaculum (1).

162. Dorsal sclerotization on A9: absent (0); present (1).

163. L group of A9: unisetose (0); bisetose (1); trisetose (2); absent (3).

164. SV group of A9: unisetose (0); bisetose (1); trisetose (2); absent (3).

165. Trichiose pubescence around anal orifice: absent (0); present (1).

166. Lateral apophyses with caudal swellings in A10: absent (0); present (1).

167. Anal comb formed as sclerotised transverse ridge with row of stout setae: absent (0); present (1).

168. Dorsal surface of anal shield: straight or slightly convex (0); concave (1).

169. Upcurved horn on caudal margin of anal shield: absent (0); present (1).

170. Posterior margin of anal shield: evenly rounded or straight (0); bifurcate (1).

171. Teeth on caudal margin of anal shield: absent (0); present (1).

**PUPAL CHARACTERS**

172. Pupal appendages: free (0); lightly fused to each other and to the body in eclosion getting free (1); firmly fused to body (2).

173. Secondary hair vestiture on pupa outside cremaster area: absent (0); secondary hairs present on ventral side of pupal abdomen (1); secondary hairs present overall (2).

174. Basic setae on pupa: absent or present as straight (0); present as distally curved (1).

175. Rough stiff setomorph pubescence on pupa outside cremaster area: absent (0); present (1).

176. Ventrally directed projection on caudal part of pupal clypeus: absent (0); present (1).

177. Ventrally or ventrodistally directed spine on cephalic part of pupal clypeus: absent (0); present (1).

178. Protuberance with concave or spoon like margins on pupal clypeus: absent (0); present (1); entirely shaped as ventrally directed beak (2).

179. Paired ventrally or ventrodistally directed protuberance on pupal clypeus: absent (0); present (1).

180. Forward directed protuberance or prolongations of varying shape on cephalic part of pupal clypeus: absent (0); present single (1); present paired (2).

181. Transverse sulcus separating vertex and frons epicranial sulcus: absent (0); present (1).

182. Frontoclypeal sulcus: absent (0); present (1).

183. Sculpted laterally extended flange on pupal eyepiece: absent (0); present (1).

184. Pupal mandibulae: not distinguishable by a presence of externally visible sulcus (0); distinguishable by a presence of externally visible sulcus (1); greatly enlarged and functional (2).

185. Pupal gena: not distinctive (0); with caudal swelling (1).

186. Pupal labium labial palpus: absent (0); present as unpaired vestigial (1); present paired (2).

187. Pupal proboscis: absent (0); present (1).

188. Cephalic extension of proboscis: not extended beyond level of eye pieces in cephalic direction (0); proboscis extended between eye pieces cephalic direction (1).

189. Base of pupal labial palpus: separate from proboscis (0); fused to pupal proboscis (1).

190. Lateral extension on pupal proboscis: absent (0); present as non-differentiated conical extension (1); present as distinctive quadrangular lobe (2); pupal proboscis and maxillary palpi fused to form a long transverse extension of proboscis (3).

191. Length of pupal proboscis: distinctly longer than labial palpus (0); about as long as or shorter than labial palpus (1).

192. Maxillary palpi: absent (0); present as triangular shaped or elongate situated laterally or laterocaudally from eye piece (1); present situated caudoventrally from eye piece (2).

193. Base of pupal antenna: not extended dorsally (0); extended dorsally to cover vertex (1).

194. Pupal flagellae: entirely separate (0); distally touching each other (1); parallelly approaching or touching each other in median or distal part thus partly invaginating mesotoracic legs (2).

195. Distal part of pupal flagellum: straight or if flagellum very long free (0); borders caudal margin of pupal forewing (1); inwards dorsad bent (2).

196. Shape of pupal flagellum: not distally broadened (0); distally broadened club shaped (1).

197. Transverse erected ridges on the scape of pupal antenna: absent (0); present (1).

198. Pupal fore coxa: prothoracic coxa visible (0); concealed (1).

199. Pupal fore femur: visible (0); concealed (1).

200. Base of pupal fore leg: cephalically extended about to the level of clypeus (0); cephalically extended partly laterad of the eye piece (1).

201. Apical parts of pupal fore leg: visible (0); concealed (1).

202. Pupal mesothoracic coxa: visible (0); concealed (1).

203. Distal part of pupal mesothoracic legs: separated (0); distally meeting each other (1); medially meeting each other distally separated (2).

204. Pupal metathoracic coxa: visible (0); concealed (1).

205. Apical parts of pupal metathoracic leg: distally exposed (0); distally concealed (1).

206. Pupal prothorax: undivided (0); almost or entirely divided by mesially overlaid vertex (1).

207. Paired tubercles in prothorax: absent (0); present (1).

208. Mesial swelling on pupal prothorax: absent (0); present (1).

209. Dorsal ridge in pupal mesothorax: absent (0); present single (1); present paired (2).

210. Lateral swelling on pupal mesothorax: absent (0); present (1).

211. Transverse row of broad punctuation on cephalic margin of pupal mesothorax: absent (0); present (1).

212. Position of opening of thoracic spiracle: externally invisible (0); between pronotum and mesonotum (1); on pronotum (2); on mesonotum (3).

213. Protuberance under opening of thoracic spiracle: absent (0); present on prothorax (1); present laterad of opening (2); present on mesothorax (3).

214. Lateral exposition of pupal hindwing: to abdominal segment 3 or more (0); at most to abdominal segment 2 (1).

215. Mesial exposition of pupal hindwing: exposed (0); concealed (1).

216. Deep impressions near caudal margin of pupal mesothorax: absent (0); pair of impressions present (1); row of impressions present (2).

217. Prominent dorsal keel on T3: absent (0); present (1).

218. Prominent dorsolateral spines on T2-3: absent (0); present (1).

219. Prominent dorsolateral spines on A1-7: absent (0); present (1).

220. Spiracle on pupal abdominal segment A1: exposed (0); concealed (1).

221. Spiracle on pupal abdominal segment A2: exposed (0); concealed (1).

222. Folding around spiracle of abdominal segment A2: absent (0); present (1).

223. Spiracle on pupal abdominal segment A3: exposed (0); concealed (1).

224. Folding around spiracle of pupal abdominal segment A3: absent (0); present (1).

225. Spiracle on pupal abdominal segment A7: of equal size to those of A2 6 (0); smaller than those of A2 6 (1).

226. Abdominal spiracles: flat on integument or at most slightly erected (0); at least some of abdominal spiracles erected so that cuticle is forming a perpendicularly protruding stalk (1); erected caudally directed (2); erected distally expanded thus fungus shaped (3).

227. Lobe on at least some abdominal spiracles: absent (0); present (1).

228. Distinctive grooves between pupal abdominal segments A1-A4 dorsally: absent (0); present (1).

229. Dorsal swelling on pupal abdominal segments A1-A4: absent (0); present (1).

230. Lateral swellings on pupal abdominal segments A2-4: absent (0); present (1).

231. Pupal abdominal intersegment 3-4: fully movable (0); limitedly movable (1); immobile (2).

232. Pupal abdominal intersegments 4-7: movable in at least one sex (0); entirely immobile (1).

233. Series of transverse furrows on caudal part of pupal abdominal segments 4-7: absent (0); present (1).

234. Regular row of spines near dorsocephalic margin of at least some segments of pupal abdomen: absent (0); present (1).

235. Irregular area of small spines in dorsomedian or cephalic part of abdominal segments: absent (0); present (1).

236. Transverse rows of laterally compressed upward directed spines near cephalic margin of abdominal segments: absent (0); present (1).

237. Transverse rows of caudocephalically compressed upward directed spines near cephalic margin of abdominal segments: absent (0); present (1).

238. Transverse rows of spines on dorsocaudal margin of at least some segments of pupal abdomen: absent (0); present (1); a few prominent discrete spines present (2).

239. Oppositely directed pairs of stout curved spines separated by transverse groove on A5-7 dorsally: absent (0); present (1).

240. Spine on at least some abdominal segments laterally adjacent to L seta: absent (0); present (1).

241. Spinose zone ventrally-ventrolaterally on pupal A5: absent (0); present (1).

242. Scars of prolegs: absent (0); present (1).

243. Lateral condyles on pupal abdominal segments limiting lateral movement of abdominal segments: absent (0); present (1).

244. Dorsal condyles on pupal abdominal segments limiting dorsoventral movement of abdominal segments: absent (0); present (1).

245. Ventral condyles on pupal abdominal segments limiting dorsoventral movement of abdominal segments: absent (0); present (1).

246. Dorsal ridge on pupal abdomen: absent (0); present (1).

247. Lateral ridge on pupal abdomen: absent (0); present (1); abdominal segments swollen laterally (2).

248. Dense row of setae on posterior margin of abdominal segment 7: absent (0); present (1).

249. Concave emargination dorsolaterally on posterior margin of abdominal segment 7: absent (0); present (1).

250. Single dorsal projection on pupal abdominal segment 8: absent (0); present (1).

251. Transverse fold at ventral side of A8 with pair of variably shaped appendices: absent (0); present (1).

252. Discernible sulcus between pupal abdominal segments 8 and 9: absent (0); present (1).

253. Discernible sulcus between pupal abdominal segments 9 and 10: absent (0); present (1).

254. Dentate groove dorsally or dorsolaterally at sulcus between A9 and A10: absent (0); present (1).

255. Shape of ventral side of pupal abdominal segments 9-10: unmodified (0); modified as a plate (1).

256. Pair of spur groups in pupal abdominal segment 9 ventrally: absent (0); present (1).

257. Pupal legs in abdominal segment 9 ventrally sometimes only present as a pair of swellings: absent (0); present (1).

258. Oval pit on pupal abdominal segment 9 laterally: absent (0); present (1).

259. Row of pores on or near pupal intersegment 9 and 10: absent (0); present (1).

260. Pair of ventrocephalically directed swellings pupal on legs on abdominal segment 10: absent (0); present (1).

261. Genital orifice invaginated in large circular groove on A10: no (0); yes (1).

262. Pair of spur groups on cephalic margin of A10: absent (0); present (1).

263. Lateral incision on pupal abdominal segment 10: absent (0); present (1).

264. Pair of dorsal spines on pupal segment 10: absent (0); present (1).

265. Lateral spine on pupal segment 10: absent (0); present (1).

266. Pair of ventral spines on pupal segment 10: absent (0); present (1).

267. Pupal cremaster: without other modifications except often a small group of setae (0); present as a setose cylindrical or dorsoventrally flattened often bifurcate appendix (1); present and consisting of paired lobes (2).

268. Pupation: concealed in cocoon soil litter foliage or within stem (0); exposed (1).

269. Presence of cocoon absent (0); present (1).

270. Structure of cocoon: simple web other material may be interwoven (0); mesh (1).

271. Shape of cocoon: undifferentiated usually oval or spindle shaped if pupation in stem cocoon simple silky chamber (0); pear shaped (1); shaped like upturned boat (2); formed of portable case of larval shape depending on the material and the way the larval case is constructed (3).

272. Longitudinal ribs on cocoon: absent (0); present (1).

273. Adult eclosion: hole of cocoon tubular or undifferentiated (0); three lobed (1); with horizontal slit (2); with transverse slit (3).

274. Lid that opens like a cap at eclosion in cocoon: absent (0); present (1).

275. Semicircular lid covering eclosion hole of cocoon: absent (0); present (1).

276. Posterior slit in cocoon: absent (0); present (1).

277. Silken girdle to hold the pupa attached to the surface around abdomen: absent (0); present (1).

278. Position of silken girdle: surrounding pupa on metathorax (0); on A1 (1); on A3 (2); on intersegment A7 A8 (3).

279. Behaviour of pupa prior to eclosion: the pupa protrudes from cocoon by active movement (0); pupa does not move out from the cocoon (1).

280. Adult eclosion: from cephalic part of cocoon (0); from caudal part of cocoon (1).

**ADULT CHARACTERS**

281. Male antenna filiform or serrate: no (0); yes (1).

282. Male filiform antennae, segments distinctly thickened in middle of antenna but taper again towards tip: no (0); yes (1).

283. Male antennae lamellate: no (0); yes (1).

284. Female antenna filiform or serrate: no (0); yes (1).

285. Female filiform antennae, segments distinctly thickened in middle of antenna but taper again towards tip: no (0); yes (1).

286. Female antenna lamellate: no (0); yes (1).

287. Distal part of flagellum: not clavate (0); distinctly thickened or clavate (1).

288. Apiculus: absent (0); present (1).

289. Apical depressions on antennal club: no (0); yes (1).

290. Longitudinal mesial groove or depression on antennal segments: no (0); yes (1).

291. Raised ventral carinae on antennal segments: no (0); yes (1).

292. Male antennae with antennal segments with two rows of sensilla on ventral side: no (0); yes (1).

293. Male antennae pectinate: no (0); yes (1).

294. Pectinate male antennae: bipectinate (0); tripectinate (1); quadripectinate (2).

295. Unipectinate male antenna: pectinate medially (0); pectinate laterally (1).

296. Pectination on antenna, male: many apical segments without well-developed rami or rami abruptly shorter at apex (0); to the tip (1).

297. Rami scaled on dorsal side, male: no (0); yes (1).

298. Tip of rami with spine: absent (0); present (1).

299. Finger like projection next to apical spine of ramus: no (0); yes (1).

300. Female antennae pectinate, often with smaller rami than males: no (0); yes (1).

301. Pectinate female antenna: bipectinate (0); tripectinate (1).

302. Female antenna: reduced (0); normal (1).

303. Antennal apex with bundle of long hairs: no (0); yes (1).

304. Antenna with long terminal segment at least four times as longer than second to apical segment: no (0); yes (1).

305. Dorsal scaling of male antennae: apex devoid of scales (0); scaled to the tip (1); no scales on dorsal side of flagellum or only at base of antenna (2).

306. Dorsal scaling of female antennae apex devoid of scales (0); scaled to the tip (1); no scales on dorsal side of flagellum or scales only at base (2).

307. Number of rows of scales on antenna: no distinct rows (0); one (1); two (2); three (3).

308. Two dorsal rows of scales, male: neither extends on the ventral side (0); only the other row of scales extends on the ventral side (1); both extend on the ventral side (2).

309. Two rows of antennal scales female neither extend on ventral side (0); the other row of scales extends on ventral side (1); both extend on ventral side (2).

310. Ventral scaling of male antennae: absent or present at base only (0); present from base to tip (1); only tip devoid of scales (2); scaleless patch on each segment on ventral side (3).

311. Ventral scaling of female antennae: absent or present at base only (0); present from base to tip (1); only tip devoid of scales (2); scaleless patch on ventral side of antennal segments (3).

312. First flagellar article of male antenna medially notched: no (0); yes (1).

313. First flagellar article 3 times longer than second segment: no (0); yes (1).

314. Base of scape: not sunken in head so that the narrower base is clearly visible (0); sunken so that the narrower base is not visible (1).

315. Antennal scape broad, partially covering eye: no (0); yes (1).

316. Eyecap formed of scales: no (0); yes (1).

317. Pecten with narrow stiff scales: absent (0); present (1).

318. Pecten with lamellate scales: absent (0); present (1).

319. Intercalary sclerite: not discernible (0); discernible (1); tiny (2).

320. Eye: not emarginate (0); emarginate adjacent to insertion of antenna (1).

321. Eye ring: absent (0); partial glossy eye ring lacking distinct ommatidial facets and widest adjacent to insertion of antenna present (1); complete eye ring with distinct ommatidial facets present (2).

322. Scales between eye and antenna: absent (0); present (1).

323. Conspicuous inter ommatidial hairs: absent (0); present (1).

324. Male eye horizontally divided by scale band, female eye partially divided by V shaped notch: no (0); yes (1).

325. Lateral scale tuft eyelash on antennal base: absent (0); present (1).

326. External lateral ocellus: absent (0); present (1); reduced (2).

327. Protrusion behind antenna some sort of reduced ocellus: absent (0); present (1).

328. Spines on vertex: absent (0); present one pair (1); present two pairs (2).

329. Distinct sensillum bearing nipples on vertex: absent (0); present (1).

330. Chaetosema: absent (0); present (1).

331. Chaetosemata: just setae (0); setae and scales (1).

332. Chaetosemata extend on base of scape: absent (0); present (1).

333. Pair of chaetosema like organs below antennae on frons: absent (0); present (1).

334. Upwardly directed lamellate scales on frons: absent (0); present (1).

335. Frontoclypeus: not protruded (0); clearly protruded (1).

336. Frontoclypeus with conspicuous central protrusion: absent (0); present round (1); present ending in a point (2).

337. Asymmetrical pits on lower part of frontoclypeus: absent (0); present (1).

338. Large movable labrum: absent (0); present (1).

339. Proboscis: absent (0); present distinctly shortened reaches at most distal tip of coxa (1); present long reaches beyond coxa often coiled (2); galea bladder like (3).

340. Scales on proboscis: absent (0); present (1).

341. Proboscis heavily sclerotized sharply pointed and armed with tearing hooks: absent (0); present (1).

342. Pilifers: absent (0); present as at least some setae (1); present as scales (2).

343. Bilobed pilifer phonoreceptor: absent (0); present (1).

344. Distinct finger like lobes next to pilifers: absent (0); present (1).

345. Maxillary palpus: absent (0); present with at most 4 segments (1); present with 5 segments (2).

346. Maxillary palpi folded on pilifers: absent (0); present (1).

347. Scales on maxillary palpi: absent (0); present (1).

348. Labial palpi: absent (0); present (1).

349. Number of segments on labial palpi 1-2 (0); 3 (1); male 3 female 2 (2).

350. Long scales on dorsal side of labial palpi brushing against eye: absent (0); present (1).

351. Lateral bristles on labial palpus: absent (0); present scattered on lateral side (1); present as tuft at distal end of second segment of labial palpus (2).

352. Chaetosema-like organ on labial palpus segment 1 lateroventral side: absent (0); present (1).

353. First labial palp segment dilated in mesial direction: no (0); yes (1).

354. Male labial palpus segment 3 with spines at tip: no (0); yes (1).

355. Vertex clearly protruded: no (0); yes (1).

356. Sulcus at back of head perpendicular to posterior edge of head: absent (0); present (1).

357. Longitudinal sulcus between transfrontal sulcus and tranverse sulcus on vertex: absent (0); present (1).

358. Patagia: absent or present at most as a tuft of scales on a small inflated structure (0); present as a distinct flap (1); present as a somewhat inflated structure with connecting part extending far down along sides of pronotum (2).

359. Pronotum without lateral extensions (0); with elongate lateral arms which extend into patagia (1).

360. Parapatagia distinct lobes: absent (0); present (1).

361. Dorsal extremities of prescutal clefts on mesothorax: relatively close clefts obliquely directed medially and the length between clefts about 1 3 of width of mesothorax (0); relatively great length between clefts over 1/2 of width of mesothorax (1); clefts meet or nearly meet medially (2); relatively close clefts continue as sutures that meet medially (3).

362. Stridulatory organ ridges on mesothorax adjacent to tegulae: absent (0); present (1).

363. Stridulatory organ small spines on mesothorax adjacent to tegulae II: absent (0); present (1).

364. Longitudinal double line on mesoscutum: absent (0); present (1).

365. Mesothoracic cuticle with darker patches: absent (0); present (1).

366. Cervical sclerites: not joined ventromedially strong reduction of ventral arms of latero cervicalia (0); joined ventromedially above sternum (1); join at sternum (2).

367. Cervical sclerites with strong inward turn: absent (0); present (1).

368. Ventral arm of laterocervicale ending abruptly mesad in a very thin rod: absent (0); present (1).

369. Strong reduction or loss of the ventral arms of the laterocervicalia: absent (0); present (1).

370. Anepisternum of mesothorax: well developed (0); reduced to a tiny sclerite or absent (1).

371. Precoxal suture: reaches anterior margin of basisternum or beyond absent (0); present (1).

372. Parepisterna sensu Brock 1971 partly concealing margin of basisternum: absent (0); present (1).

373. Parepisternal suture: absent or only partly present (0); well-developed all the way from the basisternum to the anapleural cleft (1).

374. Parepisternal suture II: strongly curved (0); running in a straight or smoothly curved line to base of spinasternum (1).

375. Secondary sternopleural suture: absent (0); present (1); partly as non-sclerotized depression (2).

376. Spinasternum with slerotized posterior part connected to basisternum: absent (0); present (1).

377. Spinasternum bifurcate: absent (0); present (1).

378. Spinasternum sclerotized between the bifurcating branches: absent (0); present (1).

379. Ventral processes of tegula: blunt (0); sharp (1); elongate spatulate (2).

380. Mesophragma: without dorsal processes (0); with dorsal processes (1); with flat ridges (2).

381. Slit on mesophragma reaches well up onto dorsal side: no (0); yes (1).

382. Slit on mesophragma with dark bar above it: no (0); yes (1).

383. Mesothorax, line of junction on meron and epimeron: short (0); long (1); midway (2).

384. Suture in shape of Y on mesepimeron: absent (0); present (1).

385. Mesopleuron with anterodorsal part of epimeron forming bag like protuberance which conceals the pleural suture: no (0); yes (1).

386. Fusion of mesothoracic lamella: not all the way up the mesofurca (0); all the way up the mesofurca (1).

387. Mesothoracic furca with lateral extensions: no (0); yes (1).

388. Fenestra media of metapostnotum: small not expanded (0); large expanded (1).

389. Metathorax fenestrae laterales: absent or reduced (0); present (1).

390. Metathoracic tympanal organs: absent (0); present (1).

391. Noctuoidea metascutal bulla: absent (0); present (1).

392. Noctuoidea counter tympana adjacent and membrane strongly pouched: absent (0); present (1); present with bottom of pouch well beyond upper rim of thoraco-abdominal cavity (2).

393. Tympanic membrane: oriented ventrally (0); oriented posteriorly (1).

394. Noctuoidea nodular sclerite: absent (0); present (1).

395. Metathoracic tymbal organs: absent (0); present (1).

396. Division of metascutum: not divided mesally (0); divided mesally (1).

397. Undivided metascutum with incision in the middle: absent (0); present (1).

398. Divided metascutum anterior margin: metascutum margin folded inward so that it crosses anterior rim of metascutellum (0); metascutum margin not folded (1); sutures of metascutum reach the metascutellum but there still is a strip of cuticle over the metascutellum (2).

399. Anteroposterior suture in the middle of metascutum: absent (0); present in both male and female (1); present in female (2).

400. Short anteroposteriorly directed suture at the base of the metascutellum: absent (0); present (1).

401. Anteriorly directed sutures from metascutellum: absent (0); present (1); present anterolaterally directed (2).

402. Metathoracic lamella anteriorly connected to coxa: no (0); yes (1).

403. Metathoracic intercoxal lamella with lateral lamellae perpendicular to it: no (0); yes (1).

404. Fusion of lamella with furca: no (0); yes (1).

405. Metathoracic furca with ventrodistal extension: no (0); yes (1).

406. Internal laminae of secondary arm of metafurca: absent (0); present (1).

407. Metathorax patch of microtrichial spines aculei on metascutum: absent (0); present (1).

408. Suture on metascutum below where microtrichiated area would be: absent (0); present (1); present and fused to anteriorly directed sutures of metascutellum (2).

409. Metathorax coxal process: absent (0); present (1).

410. Metacoxal trochantin elongated: no (0); yes (1).

411. Female posterior part of the metepimeron modified into a concave oval sclerite: absent (0); present (1).

412. Secondary arms of metafurca with pair of projections: absent (0); present (1); present as flaps (2).

413. Posterior tendons of metafurcal apohyses elongated caudad or dorsocaudad: absent (0); present (1).

414. Furcal bridge fusion of metafurca to the secondary furcal arms: absent (0); present (1).

415. Laminae between posterior tendons and secondary arms of metafurca: absent (0); present (1).

416. Metafurca with long apically fused anterior tendons: absent (0); present (1).

417. Metepimeron forming an acute angle near the posterior extremity of the subalare: absent (0); present (1).

418. Hindwing with area of small specialised scales adjacent to humeral cell: absent (0); present (1).

419. Wing venation: homoneurous (0); heteroneurous (1).

420. Narrow tailed hindwings: absent (0); present (1).

421. Female wings: absent or reduced (0); present (1).

422. Forewing with erect spicular scales: absent (0); present (1).

423. Groups of lamellar scales in forewing fringe: absent (0); present (1).

424. Forewing male stigmata i.e. patches of short specialized androconial scales on dorsal surface: absent (0); present (1).

425. Wings translucent with scales on margins and veins: absent (0); present (1).

426. Male with scent brush arising from posterior base of hindwing and resting in a particular fold of the pleura of the first and or second abdominal segments: absent (0); present (1).

427. Jugum absent (0); present (1).

428. Male subcostal retinaculum: absent (0); present as hook curled around frenulum (1); present as broad fold (2).

429. Male costal retinaculum: absent (0); present as broad based hook (1).

430. Male frenulum: absent (0); one bristle (1); several bristles (2).

431. Male frenulum with clubbed tip: no (0); yes (1).

432. Costal bristles: absent (0); present (1).

433. Female frenulum acanthi: absent (0); present as a single bristle (1); present as several stout bristles (2).

434. Female frenulum with fine hair like bristles: absent (0); present (1).

435. Female frenulum with clubbed tip: absent (0); present (1).

436. Sesiid wing linking mechanism: absent (0); present (1).

437. Harmaclonine wing locking mechanism: absent (0); present (1).

438. Costal fold: absent (0); present (1).

439. Forewing orbicular stigma: absent (0); present (1).

440. Female foreleg: not strongly reduced (0); strongly reduced (1).

441. Forecoxa: without terminal peg-like process (0); with terminal peg like process extending past trochanter at least in male (1).

442. Foretibia modified into strong apical hook: absent (0); present (1).

443. Male epiphysis: absent (0); present (1); present reduced (2).

444. Female epiphysis: absent (0); present (1); present reduced (2).

445. Number of male foreleg tarsomeres: 5 (0); 2 4 (1); 1 (2).

446. Number of female foreleg tarsomeres: 5 (0); 2 4 (1); 1 (2).

447. Short foretibia and tarsi female with first 2-3 tarsi with two very strong spines on outer side: absent (0); present (1).

448. Foreleg tarsomeres 1-4 with well-developed spines: absent (0); present (1).

449. Foreleg tarsomere five with well-developed spines: absent (0); present (1).

450. Pterothoracic legs with at least apical spines: absent (0); present (1).

451. Foreleg spines on dorsal side of tarsi: absent (0); present (1).

452. Mid leg spines on dorsal side of tarsi: absent (0); present (1).

453. Hindleg spines on dorsal side of tarsi: absent (0); present (1).

454. Female foreleg tarsomere 4 reduced and just apical pair of spines male foreleg tarsomere 4 with several spines: absent (0); present (1).

455. Female hindleg tarsi 2-4 with strong apical spine medially on ventral side: absent (0); present (1).

456. Terminal structure of male foreleg claws: absent (0); claws present (1).

457. Male foreleg claws: equal (0); unequal (1).

458. Foreleg claws serrated: absent (0); present (1).

459. Terminal structure of male foreleg lacking claws terminal segment: not downcurved to a sharp point (0); terminal segment downcurved to a sharp point (1).

460. Ventral tarsal spines on reduced male foreleg: absent (0); present (1).

461. Terminal structure of female foreleg claws: absent (0); claws present (1).

462. Claws of pterothoracic legs: curved (0); almost straight (1).

463. Claws of pterothoracic legs II: simple (0); bifid in the sense that the basal lobe is developed as a claw endodont subequal to claw (1); with basal lobe (2); bifid (3).

464. Pterothoracic legs with pulvilli: absent (0); present (1).

465. Pulvilli: simple (0); bifid (1); setae as upper lobe (2).

466. Pterothoracic legs with aroliar pad: absent/reduced (0); present (1).

467. Mid tibia with stiff scales: absent (0); present (1).

468. Hind tibia with stiff bristles: absent (0); present on dorsal surface (1); present on ventral and dorsal side (2).

469. Pretarsus apical of metathoracic leg swollen: absent (0); present (1).

470. Hind tibia and tarsi reduced particularly the males: absent (0); present (1).

471. Sub basal scale brush on mid tibia: absent (0); present (1).

472. Hind tibia of male with enlargement: absent (0); present (1); present as inverted pouch (2).

473. Foretibia with spines other than apical spines on more or less dorsal side: absent (0); present (1).

474. Midtibia with spines other than apical spines on more or less dorsal side: absent (0); present (1).

475. Hindtibia with spines other than apical spines on more or less dorsal side: absent (0); present (1).

476. Tibial spines: present on both male and female (0); present on female only (1).

477. Foretibia apical spine: absent (0); present (1); present as one very strong spine (2).

478. Midtibia strong apical spines: absent (0); present (1).

479. Hindtibia apical spines: absent (0); present (1).

480. Tibial spurs on midleg: absent (0); 1 (1); 2 (2).

481. Tibial spurs on hindleg female: absent (0); 2 (1); 3 (2); 4 (3).

482. Tibial spur length: unequal (0); equal subequal (1).

483. Place of medial spurs on hind tibia distal half (0); proximal half (1).

484. Male metatibia with androconial scales: absent (0); present (1).

485. Transformed scales in terga: absent (0); present as spiniform scales (1); present as piliform scales (2).

486. Transformed scales in terga II: present in single area in each tergum (0); present as divided areas in each tergum (1).

487. Caudal edge of terga and sterna with row of spiniform scales: absent (0); present (1).

488. Hairpencil on S2 on abdomen: absent (0); present (1).

489. Basal abdominal hair brush organ with lever: absent (0); present (1).

490. Male pleuron II with setose process: absent (0); present (1).

491. Noctuoidea counter tympanal hood: absent or greatly reduced (0); present sclerotized (1); present membranous (2).

492. Counter tympanal hood: prespiracular (0); postspiracular (1).

493. Abdominal tergum I: flat (0); domed or inflated (1).

494. Strong anterolateral extensions of tergum 2 extending below spiracle: absent (0); present on anterior side of spiracle (1); present on both sides of spiracle (2).

495. A1 of male with postspiracular conical projections of tergal origin: absent (0); present (1).

496. Posteromedially directed sclerotised ridge from lateral rod of tergum 1: absent (0); present as short ridge (1); present extended to posterior margin of tergum 1 leaving less sclerotised windows to the lateroposterior corners of tergum 1 (2).

497. Lateral extension of sternum 2: absent (0); present (1).

498. Anterolateral process of sternum 2: does not extend to the anterior edge of tergum 1 (0); extends to anterior edge tergum 1 prespiracular (1).

499. Tergosternal connection of tergal origin: absent (0); present (1).

500. Postspiracular bar: absent (0); present (1).

501. Lateral bar of A1 with broad lateral sclerotization: absent (0); present (1).

502. Sternum 2: as one sclerite (0); divided in two sclerites (1); divided into three sclerites with caudal most sternite possessing elongate lateral arms extending anteriorly (2).

503. Abdominal tympanal organs on sternum 2: absent (0); present (1).

504. Abdominal tympana sexually dimorphic: absent (0); present (1).

505. Abdominal tympana with ansa: absent (0); present (1).

506. Abdominal tympana with spiniform process: absent (0); present (1).

507. Apodeme in sternum 2: absent (0); present (1); present as sclerotized tympanal structures at least in female (2).

508. Anterior region of sternum A2 bears a pair of large elongate transparent windows presumably tympana and a pseudo praecinctorium: absent (0); present (1).

509. Abdominal tympana praecinctorium: absent (0); present simple (1); present bilobed (2).

510. Male sternum 2 with venulae: absent (0); present (1).

511. Sternum 2 venula V shaped configuration: absent (0); present (1).

512. Secondary venulae: absent (0); present with tip pointing inward (1); present with tip pointing outward (2).

513. Sternum 2 with enlargement of sternal apodeme: absent (0); present (1).

514. Basal abdominal sternite with two internal longitudinal flanges: absent (0); present (1).

515. Transverse costa behind anterior margin of sternum 2: absent (0); present (1); present on female only (2).

516. Pair of pocket like organs on spiracle of A7: absent (0); present (1).

517. male tergum VIII forming a hood over genitalia: absent (0); present (1).

518. Male segment VIII with tergum and sternum anteriorly fused forming a slender looped structure: absent (0); present (1).

519. Posterior expansion of pleuron VIII pleural lobes: absent (0); present (1).

520. Female abdominal segment 7 8 expanded dorsally: absent (0); present (1).

521. Male S8 with patch of sex scales: absent (0); present (1).

522. Male abdominal S8: not elongate (0); sclerotized and elongate (1).

523. Slender pair of ventral pseudapophyses within A10 in female: absent (0); present (1).

524. Female post abdomen anterior apophyses: absent/reduced (0); present (1).

525. Transverse bar connecting anterior apohyses on dorsal side: absent (0); present (1).

526. Female post abdomen anterior apophysis at base continued into dorsal and ventral costae: absent (0); present (1).

527. Female posterior apophyses: absent or strongly reduced (0); present (1).

528. Ovipositor lobes fused dorsally into a single bent disc with strong setae confined to the sclerotized rim: absent (0); present (1).

529. Ovipositor quadrilobate: absent (0); present (1).

530. Papillae anales sclerotized and pointed to form piercing apex: absent (0); present (1).

531. Pair of eversible pheromone glands associated with the anal papillae of females dorsal side: absent (0); present (1).

532. Length of ovipositor: not telescopic (0); telescopic (1).

533. Papillae anales flat: absent (0); present (1).

534. Female reproductive system: monotrysian (0); exporian (1); ditrysian (2).

535. Eversible gland on dorsal side on papillae anales: absent (0); present (1).

**References:**

Chrétien, P. (1916) Observations sur la “Cimelia margarita” Hb. *Lépidoptérologie comparée* (1916) 37-65.

Common, I. F. B. (1990) *Moths of Australia*. 535 pp. Carlton, Melbourne University Press.

Davis, D. R. & Milstrey, E. G. 1988. Description of *Acrolophus pholeter*, (Lepidoptera: Tineidae) a new moth commensal from gopher tortoise burrows in Florida. Proceedings of the Entomological Society, Washington, 90, 164-178.

Davis, D. R. & Robinson, G. S. The Tineoidea and Gracillarioidea. Pp. 91—117 in N. P. Kristensen, editor. Handbook of Zoology: Lepidoptera, moths and butterflies. Vol.1: Evolution, Systematics and Biogeography. Walter de Gruyter, Berlin

Dugdale, J. S., Kristensen, N. P., Robinson, G. S. & Scoble, M. J. (1998) The smaller Microlepidoptera-grade superfamilies, pp. 217–232. In Kristensen, N. P. (ed.) Lepidoptera: Moths and butterflies. Volume 1: Evolution, systematics, and biogeography. Handbuch der Zoologie/Handbook of Zoology. Band/Volume IV Arthropoda: Insecta. Walter de Gruyter GmbH. & Co. Berlin & New York.

Epstein, M.E. (1996) Revision and phylogeny of the limacodid-group families, with evolutionary studies on slug caterpillars (Lepidoptera: Zygaenoidea). *Smithsonian Contributions in Zoology*, 582, 1-102.

Epstein, M., & Brown, J. W. (2003) Early stages of the enigmatic *Prodidactis mystica* (Meyrick) with comments on its new family assignment (Lepidoptera: Prodidactidae). *Zootaxa*, 247, 1–16.

Fänger, H., Yen, S.-H. & Naumann, C. (1998) External morphology of the last instar larva of *Phauda mimica* Strand, 1915 (Lepidoptera: Zygaenoidea). Insect Systematics and Evolution, 29, 429-450.

Hasenfuss, I, (1980) Die präimaginalstadien von *Thyris fenestrella* Scopoli (Thyrididae, lepidoptera). Bonner Zoologische Beiträge, 31, 168-190.z

Heikkilä, M., Kaila, L., Mutanen, M., Peña, C., Wahlberg, N. 2012. Cretaceous origin and repeated tertiary diversification of the redefined butterflies. Proc. R. Soc. Biol. Sci. Ser. B 279, 1093–1099.

Heikkilä. M., Mutanen, M., Kekkonen, M. & Kaila, L. (2014) Morphology reinforces proposed molecular phylogenetic affinities: a revised classification for Gelechioidea (Lepidoptera). *Cladistics*¸ e-publication ahead of print. doi. 10.1111/cla.12064

Hinton, H. E. (1946) On the homology and nomenclature of the setae of lepidopterous larvae, with some notes on the phylogeny of the Lepidoptera. Transactions of the Royal Entomological Society of London, 97, 1-37.

Horak, M., Day, M. F., Barlow, C., Edwards, E. D., Su, Y. N & Cameron, S. L. (2012) Systematics and biology of the iconic Australian scribbly gum moths *Ogmograptis* Meyrick (Lepidoptera: Bucculatricidae) and their unique insect-plant interaction. *Invertebrate Systematics*, 26: 357-398.

Kaila, L. & Sugisima, K. 2011. Phylogeny, subfamily definition and generic classification. Elachistine moths of Australia (Lepidoptera: Gelechioidea: Elachistinae). CSIRO Publishing, Melbourne, pp. x+443 pp.

Komai, F., Yoshiyasu, Y., Nasu, Y., Saito, T. 2011 (Eds.), A Guide to the Lepidoptera of Japan. Tokai University Press, Kanagawa, pp. i-xx, 1–1305.

Kristensen, N.P. 2003. Skeleton and muscles: adults. Pp. 39—122 in N. P. Kristensen, editor. Handbook of Zoology IV. Lepidoptera, moths and butterflies, Vol. 2: Morphology, physiology, and development. Berlin, New York. Walter de Gruyter.

Kyrki, J. 1983. Adult abdominal sternum II in ditrysian tineoid superfamilies – morphology and phylogenetic significance (Lepidoptera). Annales Entomologici Fennici 49, 89-94.

Minet, J. 1983. Étude morphologique et phylogénétique des organes tympaniques des Pyraloidea. 1 – généralités et homologies (Lep. Glossata). *Annales de la Sociéte entomologique France* 19, 175-207.

Minet, J. (1986) Ebauche d’une classification moderne de l’ordre des Lépidoptères. *Alexanor*, 14, 291-313.

Minet, J. 1991. Tentative reconstruction of the ditrysian phylogeny (Lepidoptera, Glossata). *Entomologica Scandinavica* 22(1): 69-95.

Minet, J. 2002. The Epicopeiidae: phylogeny and a redefinition, with the description of new taxa (Lepidoptera: Drepanoidea). Annales de la Société entomologique France 38, 463-487.

Mosher, E. (1916) A classification of the Lepidoptera based on characters of the pupa. Bulletin of the Illinois State Laboratory of Natural History, 12(2), 17-151 + 22 plates.

Nakamura, M. (2006) Pupae of Japanese Epicopeiidae. Japan Hetrocerists’ Journal, 241, 288-290.

Patočka, J. & Turčani, M. 2005. Lepidoptera pupae. Central European species. Apollo Books, Stenstrup.

Scoble, M. 1992. The Lepidoptera: form, function, and diversity. Natural History Museum in association with Oxford University Press. Oxford. 1-404.

Stehr, F. W. 1987. (ed.) Immature Insects, 1. Dubuque, Iowa. Kendall/Hunt.

Tschistjakov, Yu. A. & Belyaev, E. A. (1987). The immature stages of *Pterodecta felderi* (Bremer) and systematic position of the family Callidulidae (Lepidoptera). Tinea 12 (supplement): 285-289.

Vegliante, F. & Zilli, A. (2004). Larval morphology of *Heterogynis* (Lepidoptera: Heterogynidae). European Journal of Entomology 101: 165-184.

Vegliante, F. (2005) Larval head anatomy of *Heterogynis penella* (Zygaenoidea, Heterogynidae), and a general discussion of caterpillar head (Insecta, Lepidoptera). *Acta Zoologica*, 886, 167-197.

Yen, S.-H. & Horie, K. (1997) *Pryeria sinica* (Lepidoptera, Zygaenidae), a newly discovered relic in Taiwan. Transactions of the Lepidopterological Society of Japan, 48, 39-48.

Yen, S.-H., Mu, J.-H. & Jean, L.-J. (1995) The life histories and biology of Epicopeiidae of Taiwan. Transactions of the Lepidopterological Society of Japan, 46, 175-184.
