# Supplementary figures and images for: Elusive ditrysian phylogeny: an account of combining systematized morphology with molecular data (Lepidoptera)
Source: BMC Evol Biol. 2015 Nov 21;15:260. doi: 10.1186/s12862-015-0520-0 (PMC4654798; doi:10.1186/s12862-015-0520-0)

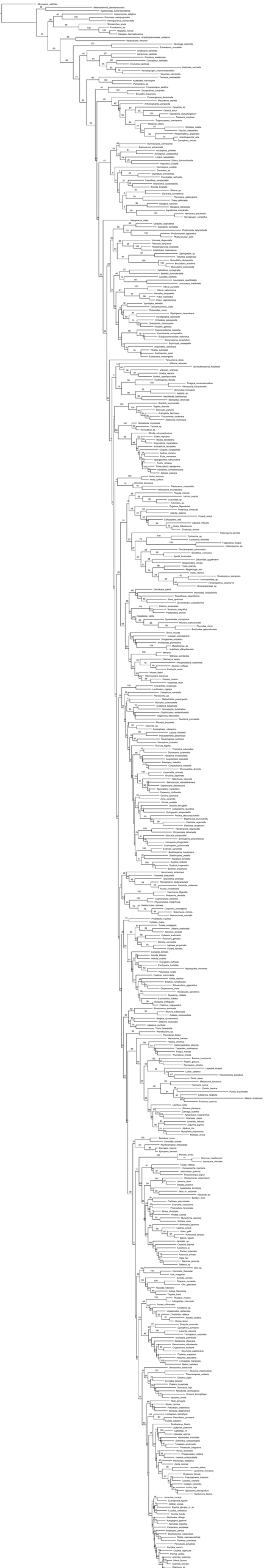

Supplement: Additional file 7: — Phylogenetic tree from maximum likelihood analysis of combined data with third codon position removed from molecular data except from EF1a. 473 taxa, 4991 characters (4461 bp, 530 morphological characters). (PDF 35 kb) [file 12862_2015_520_MOESM7_ESM.pdf]

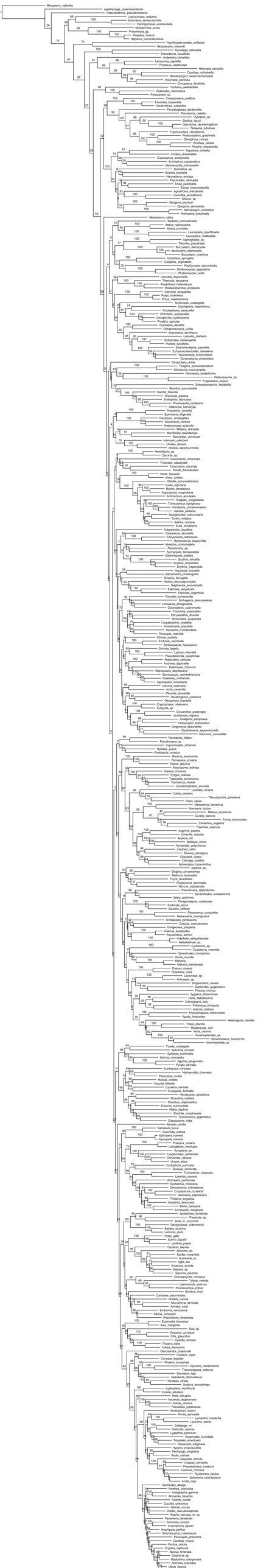

Supplement: Additional file 8: — Phylogenetic tree from maximum likelihood analysis of DNA data with third codon position retained, 422 taxa, 6172 bp. (PDF 32 kb) [file 12862_2015_520_MOESM8_ESM.pdf]

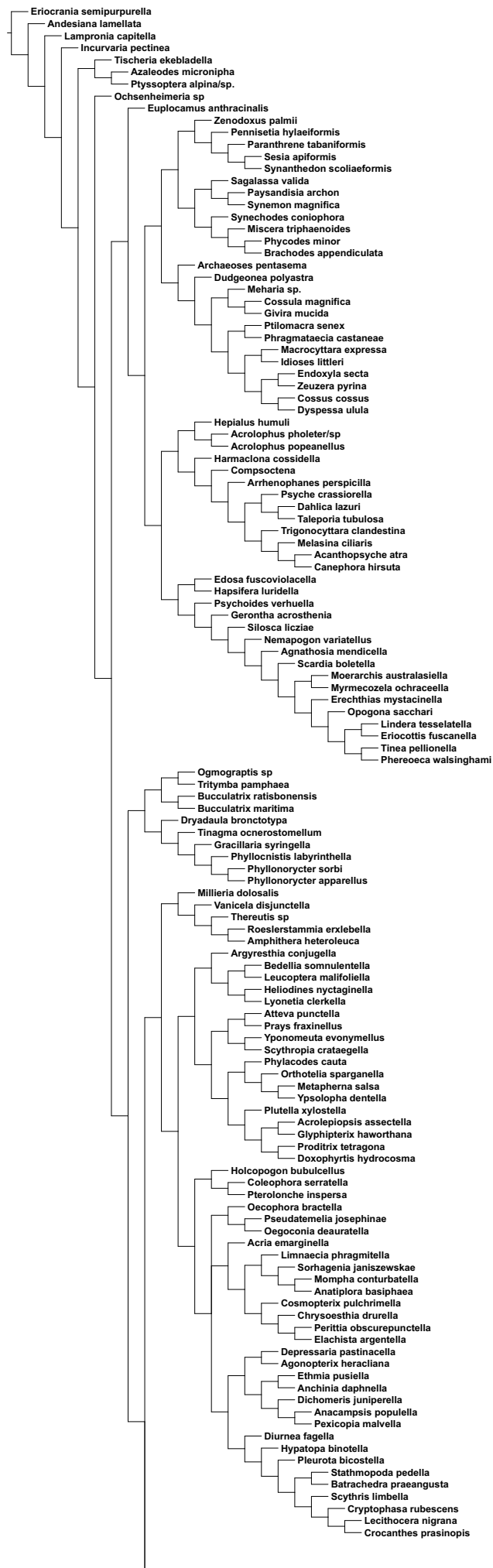

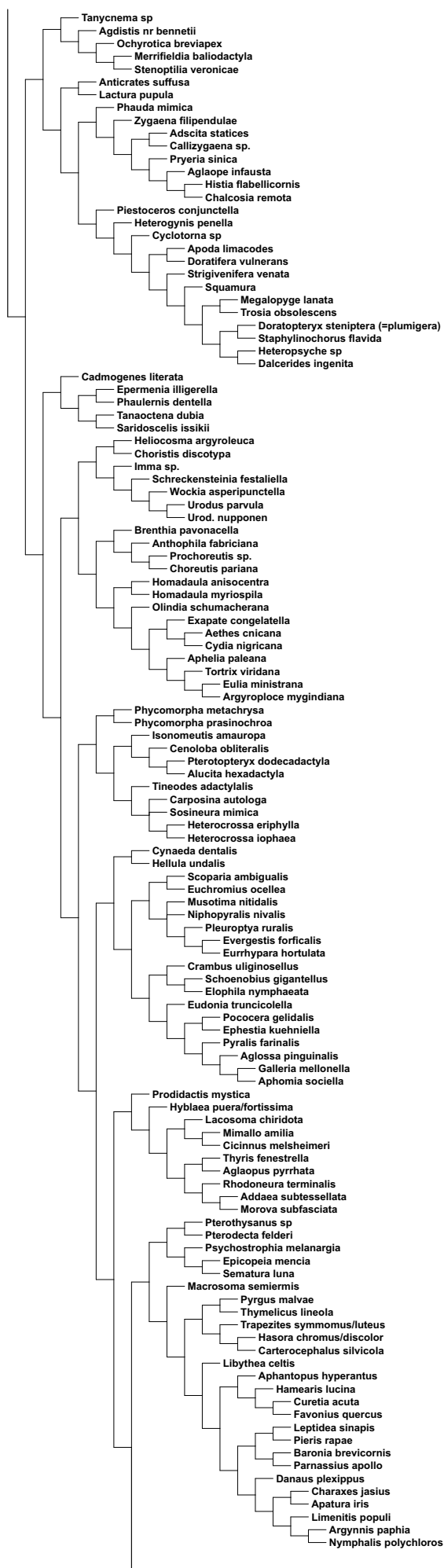

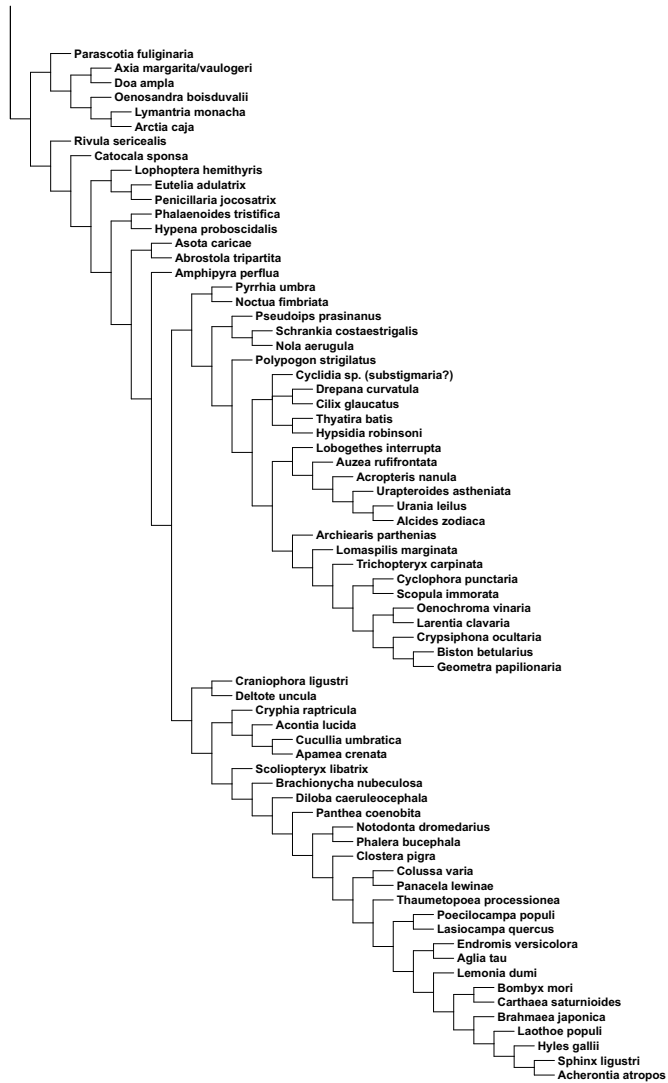

Supplement: Additional file 10: — One of the 19 equally most parsimonious trees (length = 5305 steps, Ci 12, Ri 62) from the parsimony analysis of morphological data, 318 taxa, 530 characters. Additional file 1 lists morphological data for 320 taxa, but in the present analysis, data for two species of Thereutis were concatenated into one terminal taxon as were those of two species of Cyclotorna making the total number of taxa in the analysis 318. (PDF 4061 kb) [file 12862_2015_520_MOESM10_ESM.pdf]

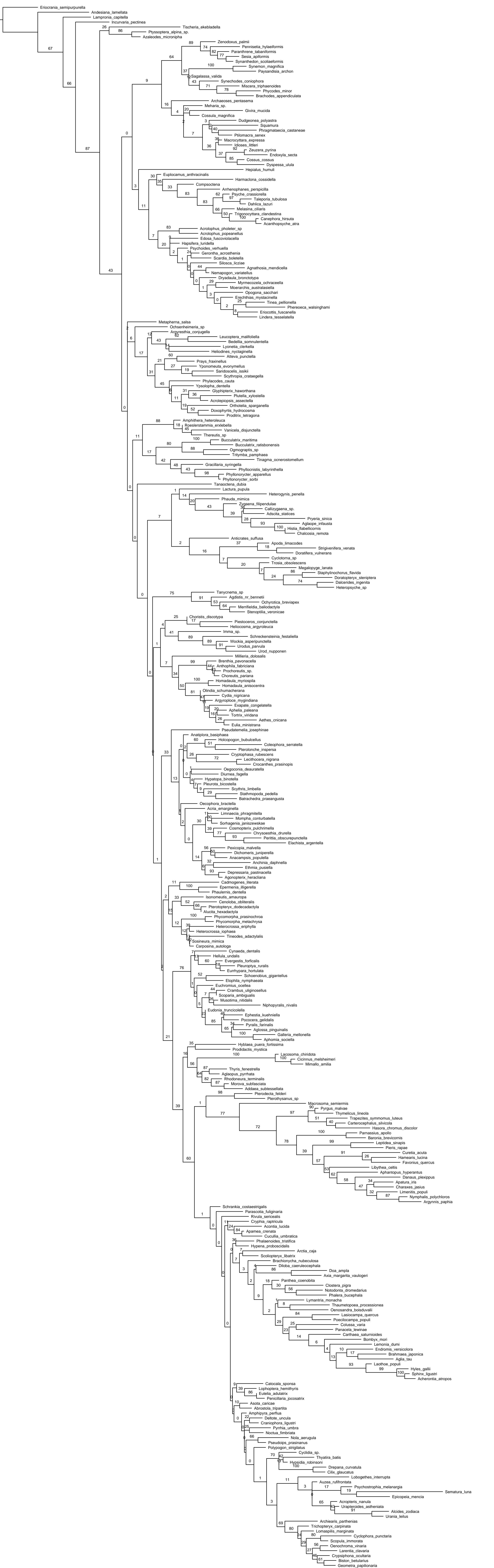

0.08

Supplement: Additional file 11: — Phylogenetic tree from maximum likelihood analysis of morphology, 318 taxa, 530 characters. Additional file 1 lists morphological data for 320 taxa, but in the present analysis, data for two species of Thereutis were concatenated into one terminal taxon as were those of two species of Cyclotorna making the total number of taxa 318. (PDF 21 kb) [file 12862_2015_520_MOESM11_ESM.pdf]

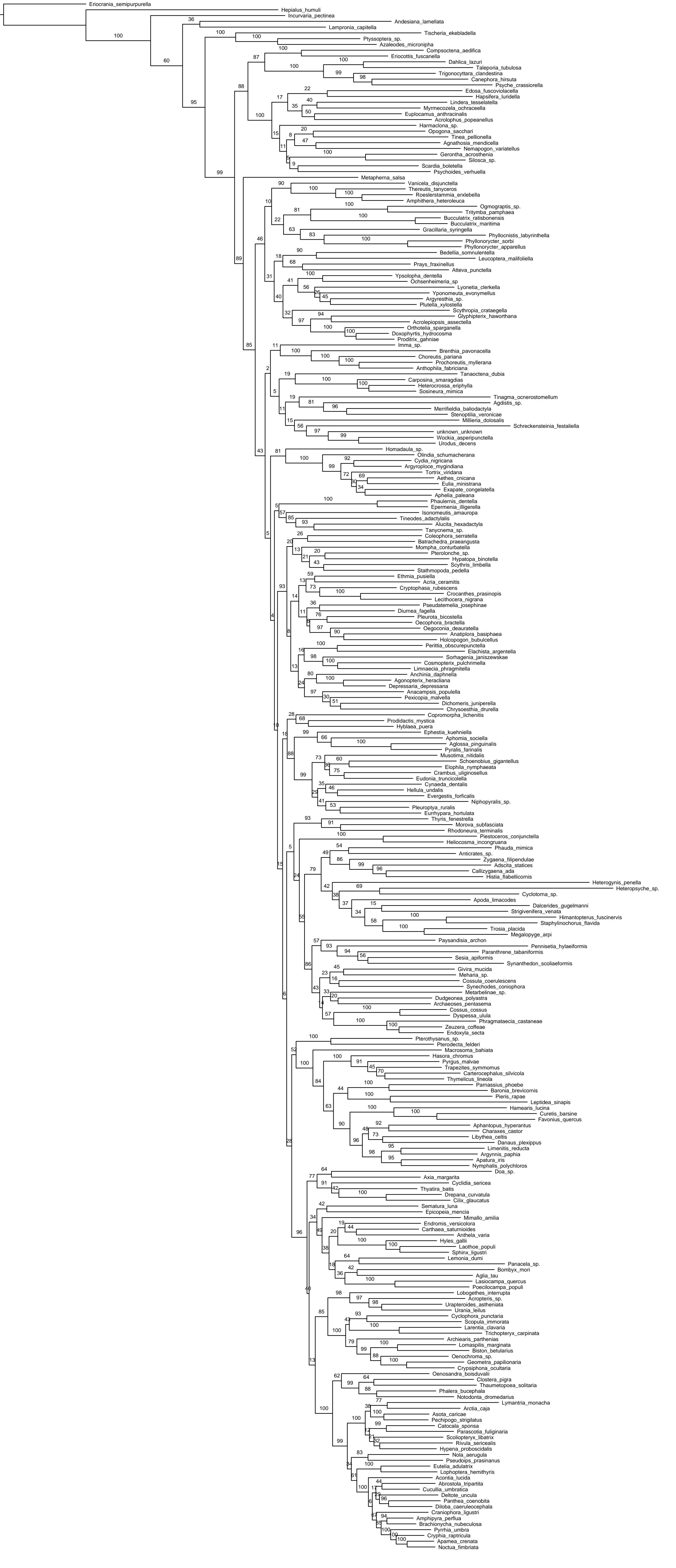

Supplement: Additional file 12: — Phylogenetic tree from maximum likelihood analysis of combined morphological and molecular data, 268 taxa. The data set includes all the taxa for which both morphological and molecular data were available. (PDF 18 kb) [file 12862_2015_520_MOESM12_ESM.pdf]

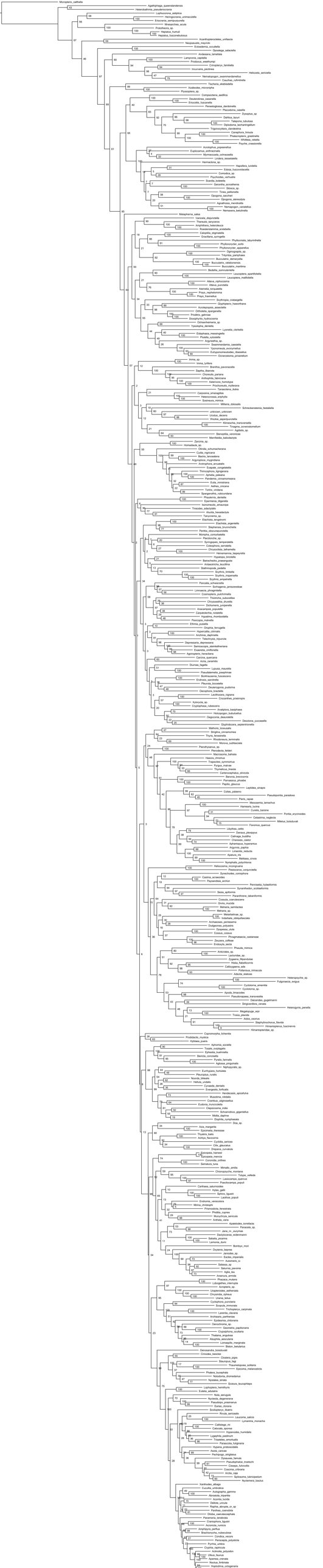

Supplement: Additional file 13: — Phylogenetic tree from maximum likelihood analysis of combined morphological and molecular data, 422 taxa. The data set includes all the taxa for which DNA data were available. Morphological data were available for 268 of these taxa. (PDF 28 kb) [file 12862_2015_520_MOESM13_ESM.pdf]
