# Supplementary material for: Elusive ditrysian phylogeny: an account of combining systematized morphology with molecular data (Lepidoptera)
Source: BMC Evol Biol. 2015 Nov 21;15:260. doi: 10.1186/s12862-015-0520-0 (PMC4654798; doi:10.1186/s12862-015-0520-0)
Supplement: Additional file 9: — Phylogenetic tree from maximum likelihood analysis of combined morphological and molecular data, 473 taxa. Molecular data were categorized according to the rate of evolution with the program Tiger and partitioned with RatePartitions. Two fastest evolving partitions (partition 7 with 482 bp and partition 6 with 357 bp) removed. (PDF 33 kb) [file 12862_2015_520_MOESM9_ESM.pdf]

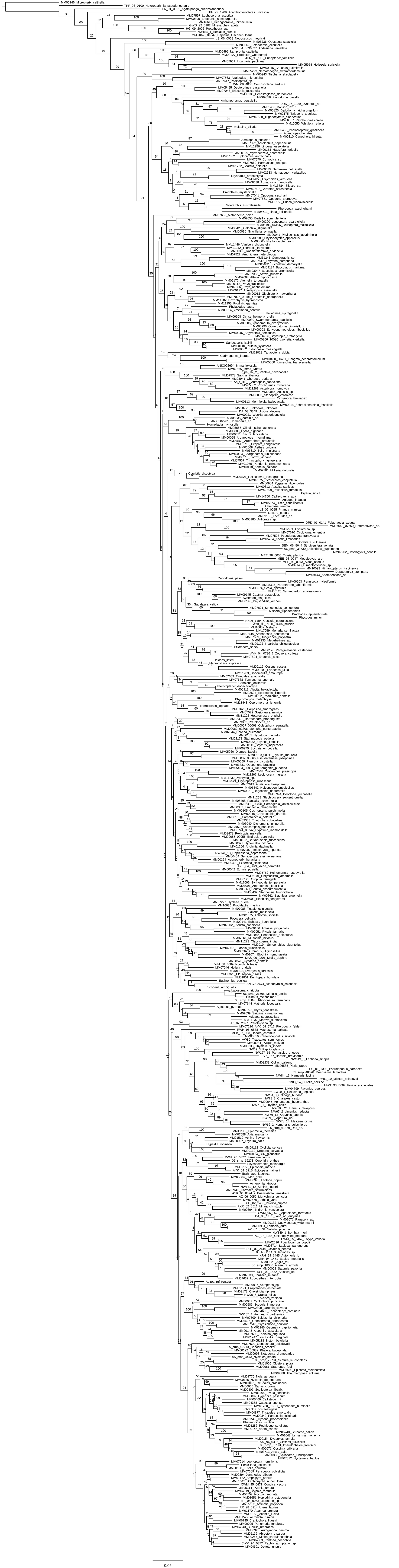
0.05
